# Analytical Framework to Understand the Origins of Methyl Side-Chain Dynamics in Protein Assemblies

**DOI:** 10.1021/jacs.3c12620

**Published:** 2024-03-13

**Authors:** Kai Zumpfe, Mélanie Berbon, Birgit Habenstein, Antoine Loquet, Albert A. Smith

**Affiliations:** † Institute for Medical Physics and Biophysics, Leipzig University, Härtelstraße 16-18, 04107 Leipzig, Germany; ‡ University of Bordeaux, CNRS, Bordeaux INP, CBMN, UMR 5248, IECB, 33600 Pessac, France

## Abstract

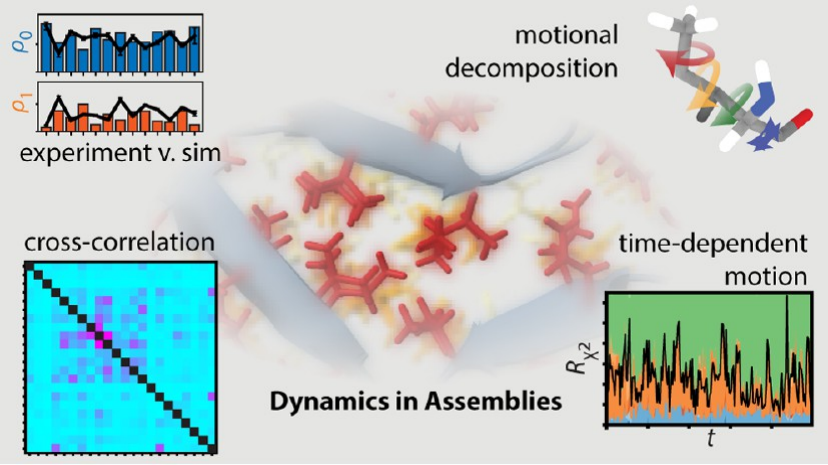

Side-chain motions play an important role in understanding protein structure, dynamics, protein–protein, and protein–ligand interactions. However, our understanding of protein side-chain dynamics is currently limited by the lack of analytical tools. Here, we present a novel analytical framework employing experimental nuclear magnetic resonance (NMR) relaxation measurements at atomic resolution combined with molecular dynamics (MD) simulation to characterize with a high level of detail the methyl side-chain dynamics in insoluble protein assemblies, using amyloid fibrils formed by the prion HET-s. We use MD simulation to interpret experimental results, where rotameric hops, including methyl group rotation and χ_1_/χ_2_ rotations, cannot be completely described with a single correlation time but rather sample a broad distribution of correlation times, resulting from continuously changing local structure in the fibril. Backbone motion similarly samples a broad range of correlation times, from ∼100 ps to μs, although resulting from mostly different dynamic processes; nonetheless, we find that the backbone is not fully decoupled from the side-chain motion, where changes in side-chain dynamics influence backbone motion and vice versa. While the complexity of side-chain motion in protein assemblies makes it very challenging to obtain perfect agreement between experiment and simulation, our analytical framework improves the interpretation of experimental dynamics measurements for complex protein assemblies.

## Introduction

Side chains play critical roles in determining protein characteristics and are central to the so-called “protein folding problem”. The chemical structure of side chains and their arrangement along the polypeptide backbone are major factors in establishing the native protein structural fold, establishing quaternary interactions in macromolecular complexes, and furthermore selecting potential interaction partners. In the context of supramolecular protein assemblies such as protein fibers, amyloid fibrils, and helical filaments, side-chain–side-chain packing and side-chain interdigitation are often the main driving forces that tune the assembly mechanisms.^1−3^ Recent developments in cryoelectron microscopy have uncovered the structural fold at atomic resolution of various pathological amyloid fibrils. For example, in the case of Tau-paired helical filaments, hydrophobic clusters made of valine, leucine, and isoleucine provide rigid and specific hydrophobic side-chain packing, allowing the supramolecular stability observed for these amyloid fibrils.^4^ In amyloid-forming peptides, steric zippers can also be stabilized by rigid side-chain interdigitation.^5^

However, structures alone do not represent the full story of side chains in fibrils. The dynamics of the side chains both play a role in forming and stabilizing the protein’s structure and determining its function via specific interactions and contributions to the protein’s entropy.^6−8^ Interplay between dynamics and side-chain packing can play an important role in protein stability. For example, packing that is too tight, restricting methyl rotation, can reduce structural stability by reducing entropic contributions from methyl libration, and in fact, structural quality can be evaluated based on estimates of the methyl rotation barrier, where high barriers indicate an unlikely structure.^9^ Furthermore, methyl rotation is activated with increasing temperature,^10^ where contributions to structural stability from the methyl groups are primarily enthalpic at lower temperatures but entropic at higher temperatures. To further complicate matters, protein “breathing” modulates the tightness of protein packing, allowing in some proteins the occurrence of aromatic ring flips;^11^ breathing motion then may also modulate other dynamics. In fibrils, the protein backbone is usually highly rigid, so that one might also assume that side chains remain mostly in one configuration. However, experimental evidence suggests otherwise: in Aβ_1–40_ amyloid fibrils, while the backbone is quite rigid, not only do methyl dynamics contribute to stability, but side chains also exhibit rotameric dynamics, including in the fibril core, as evidenced by H–C dipolar order parameters,^12^ deuterium lineshapes,^13^ and longitudinal relaxation.^14^

**Figure 1:**
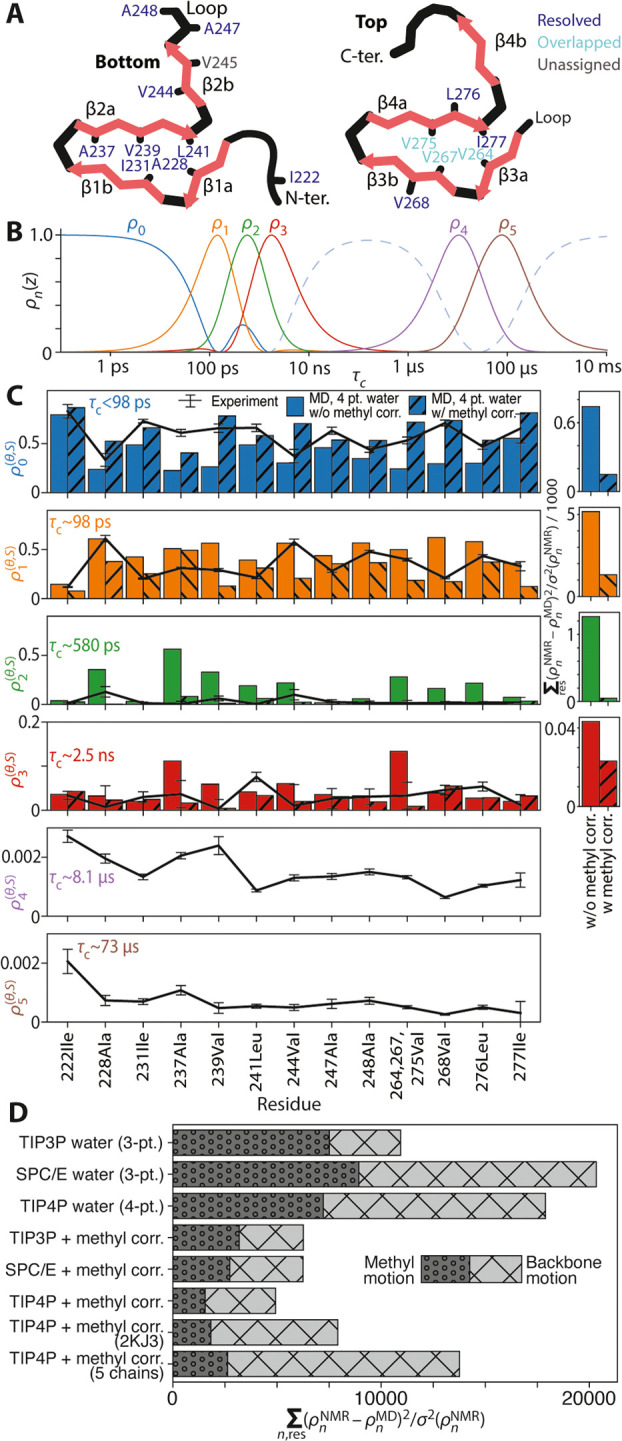
Experimental vs simulated detector responses. (A) Cartoon representation of the HET-s(218–289) structure (PDB ID 2KJ3), with Ala, Leu, Ile, and Val labeled. Residues in dark blue are spectrally resolved, light blue is assigned but overlapping, and V245 (gray) is not unambiguously assigned. Panel (B) shows sensitivities of 6 experimental detectors. Panel (C) shows experimental detector responses (black lines, error bars show ±1σ), with molecular dynamics (MD) detector responses for two simulations (open bars: 4-point water with the default methyl rotation barrier; // hatching: 4-point water with corrected methyl barrier). MD-derived detector responses are not calculated for ρ_4_–ρ_5_ since the trajectories are not long enough. Panel (D) shows the total error for the methyl and backbone (^15^N) detector responses for eight different MD simulations. The total error is divided into contributions from methyl motion (H–C, “o” hatching) and backbone (H–N, “x” hatching) motion.

In particular, clusters of hydrophobic side chains, e.g., alanine, valine, threonine, leucine, and isoleucine, where no strong electrostatic effects or π-stacking occur, may exhibit significant motion even when contained by a relatively rigid protein backbone structure. This is the case in the fibrillar amyloid architecture formed by the prion protein HET-s(218–289), where one molecule of HET-s forms two winding layers of the fibril, with residues 226–246 and 262–282, each forming four β-sheets which are connected by a flexible loop (247–261) (Figure 1A).^15,16^ These eight β-sheets have previously been shown to be highly rigid, with most backbone motion being attributed to low-amplitude collective dynamics.^17^ On the other hand, residues in the fibril core undergo methyl and rotameric dynamics. Previous NMR studies have characterized such side-chain motions for other proteins, sometimes only with an order parameter, *S*^2^, which provides insight into rotameric populations and entropic contributions from side chains^8,18^ and also with several amplitudes and correlation times for ubiquitin.^19^ However, the physical significance of the fitted correlation times, or the dependence of side-chain dynamics on the local protein packing, can still be investigated in greater detail.

Here, we investigate what factors influence side-chain motion based on a combination of nuclear magnetic resonance (NMR) relaxation data and molecular dynamics (MD) simulation^20−25^ while using advanced analysis techniques. We compare results from molecular dynamics (MD) simulation to an analysis of nuclear magnetic resonance (NMR) relaxation data^20−25^ in order to determine the degree of accuracy of the side-chain motion in the MD simulations. We then use MD simulation as a guide to interpret some of the experimental data, resulting in the characterization of methyl group rotational correlation times, methyl librational amplitudes, side-chain rotameric populations, and time scales of rotameric motion. Finally, we use analysis of the MD simulation to determine what factors influence the side-chain motion. We conclude that side-chain motion depends on several factors: side-chain configuration, interaction with nearby side chains, and mode dynamics of the protein backbone. These factors are also time-dependent so that the side-chain motional characteristics are constantly changing. We find that side-chain dynamics have an indirect impact on backbone dynamics, allowing us to improve the agreement between backbone motion in simulation and experiments by modifying side-chain parameters in the MD force field.

## Results and Discussion

### Experimental vs Simulated Results

We have measured ^13^C *T*_1_ and ^1^H–^13^C nuclear Overhauser effect (NOE) rate constants at three magnetic fields (400, 600, 700 MHz ^1^H frequencies), ^13^C *T*_1ρ_ with 5 kHz MAS and spin-lock strengths of 12, 14, and 19 kHz (600 MHz ^1^H frequency), and measured the ^1^H–^13^C residual dipole coupling with DIPSHIFT^26^ on a specifically labeled sample of HET-s(218–289) fibrils. One methyl group each for leucine, isoleucine, valine, and alanine residues was ^13^C-labeled, where the methyl hydrogens were labeled with one ^1^H and two ^2^H (^13^C^1^H_1_^2^H_2_ or ^13^CHD_2_ labeling), such that methyl ^13^C relaxation is dominated by the ^1^H–^13^C dipole coupling with weaker contributions from the ^13^C chemical shift anisotropy (CSA) and ^2^H–^13^C couplings. All experiments were performed at 300 K. Details of the sample preparation are found in SI Section S1, and experimental parameters are given in SI Section S2. Data was processed with the detector analysis,^27,28^ which captures the amplitude of reorientational dynamics for the NMR interactions within well-defined, time scale-specific windows. To explain the detector approach, we must first introduce the rank-2 reorientational correlation function, which determines NMR relaxation behavior, given by
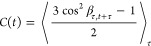
1β_τ,*t*+τ_ is the angle between the NMR interaction tensor at time τ and at a later time *t* + τ, where brackets average over the initial time, τ. *C*(*t*) has an initial value (*t* = 0) of 1 and decays to *S*^2^; multiple motions may influence *C*(*t*), resulting in complex functional behavior. However, it can generally be assumed to take the form of a sum over many decaying exponential terms, i.e.,
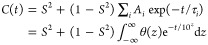
2(1 – *S*^2^) is the total amplitude of decay, and *A_i_* partition that decay over an arbitrary number of correlation times, τ*_i_* (∑*_i_A_i_* = 1). The latter expression is equivalent but expresses the partition as a distribution, θ(*z*), given on a logarithmic scale (*z* = log_10_(τ/s),∫θ(*z*)d*z* = 1).

A full parametrization of the reorientational correlation function is not possible, but we may perform a detector analysis, which returns the amplitudes of motion, i.e., detector responses, within several time scale-specific “windows”, i.e., detector sensitivities, where for a detector sensitivity, ρ_*n*_(τ), the detector response, ρ_*n*_^(θ,*S*)^ is given by
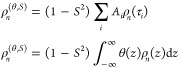
3

Sensitivities in this study are shown in Figure 1B, where ρ_1_–ρ_5_ have well-defined centers spanning from ∼100 ps to 80 μs. ρ_0_ captures motion falling outside the other windows, although, for methyl groups, ρ_0_^(θ,*S*)^ results mainly from fast methyl rotation occurring with correlation times <∼100 ps. Detector responses do not yield the exact form of θ(*z*) but return unbiased parameters describing θ(*z*), which may easily be compared to other methods, such as MD, where more detailed analyses are available.

Figure 1C shows experimental detector responses (black lines), where amplitudes result from all motions reorienting methyl H–C bonds, but three-site hopping of the methyl group makes the largest contribution (the total amplitude from methyl hopping must be 8/9; see SI Section S6). Most of this amplitude is within the time scale windows of ρ_0_ and ρ_1_, with ρ_0_ > ρ_1_, indicating a fast (τ_c_ < 100 ps) methyl hopping rate for most residues (similar results were obtained for a detector analysis of ubiquitin methyl groups^29^). The remaining detector amplitude comes from other motions, primarily 3-site hopping around the χ_1_ angle (around the Cα–Cβ bond in Val, Leu, Ile) and χ_2_ angle (around the Cβ–Cγ bond in Leu, Ile).^19,30^ Smaller librational motions and Cα–Cβ reorientation also contribute.

To obtain a more exact interpretation of the detector analysis, we use MD simulation, where the first step is to validate the quality of the MD simulation. Bars in Figure 1C compare detector responses obtained with 2 μs MD simulations of HET-s fibrils (PDB entry 2RNM) using the AMBER ff99SB*-ILDN force field^31^ and four-point (TIP4P) water, without and with correction of the methyl rotation barrier as implemented by Hoffmann et al.^32,33^ Each simulation was run at 300 K, to match the experimental conditions (additional details are found in SI Section S5). Simulations use a hydration level of 1:9 protein:water (m/m) to ensure that HET-s molecules cannot interact with themselves across the periodic boundary condition and are well-hydrated. Only detectors ρ_0_–ρ_3_ are compared to MD data since the reasonable estimation of ρ_4_ (∼8 μs) requires ∼40 μs simulation. Indeed, the MD detector responses are more in line with experimental values when using the corrected energy barrier, with improvement for all detectors (ρ_0_–ρ_3_, Figure 1C, right). We furthermore tested several other combinations of force fields and conditions, including two 3-point water models (TIP3P^34^ and SPC/E^35^) with and without methyl barrier correction, 4-point water simulations starting from the PDB entry 2KJ3,^16^ instead of 2RNM,^15^ and simulations including 3 HET-s subunits (i.e., 6 fibril layers) vs 5 subunits (i.e., 10 layers), for a total of eight simulations. The total disagreement (sum of squares, normalized by the experimental standard deviation) between the experiment and simulation is plotted in Figure 1D for each simulation (summed over all residues and ρ_0_–ρ_3_). While we focus on methyl dynamics in this study, we also compared detectors obtained for backbone H–N dynamics to experimental data.^36^ Interestingly, although one might expect the highly rigid backbone amyloid core of HET-s to be unaffected by changes in the somewhat distant methyl motion, we found that this is not the case. Indeed, for each water model, using the methyl-corrected force field also improves dynamics reproduction in the backbone; this improvement is significant for TIP4P and SPC/E water models, although less pronounced for TIP3P where backbone performance is already quite good (also see SI Figure S6). This is critical: seemingly minor changes in the force field improve the overall dynamics reproduction, indicating indirect influences between motions distant from each other. Note that unless otherwise noted, subsequent MD analyses use a 10 μs trajectory with TIP4P water, methyl correction, and 3 copies of HET-s subunit starting from the 2RNM PDB.

### Separating Motion Using MD Data

**Figure 2:**
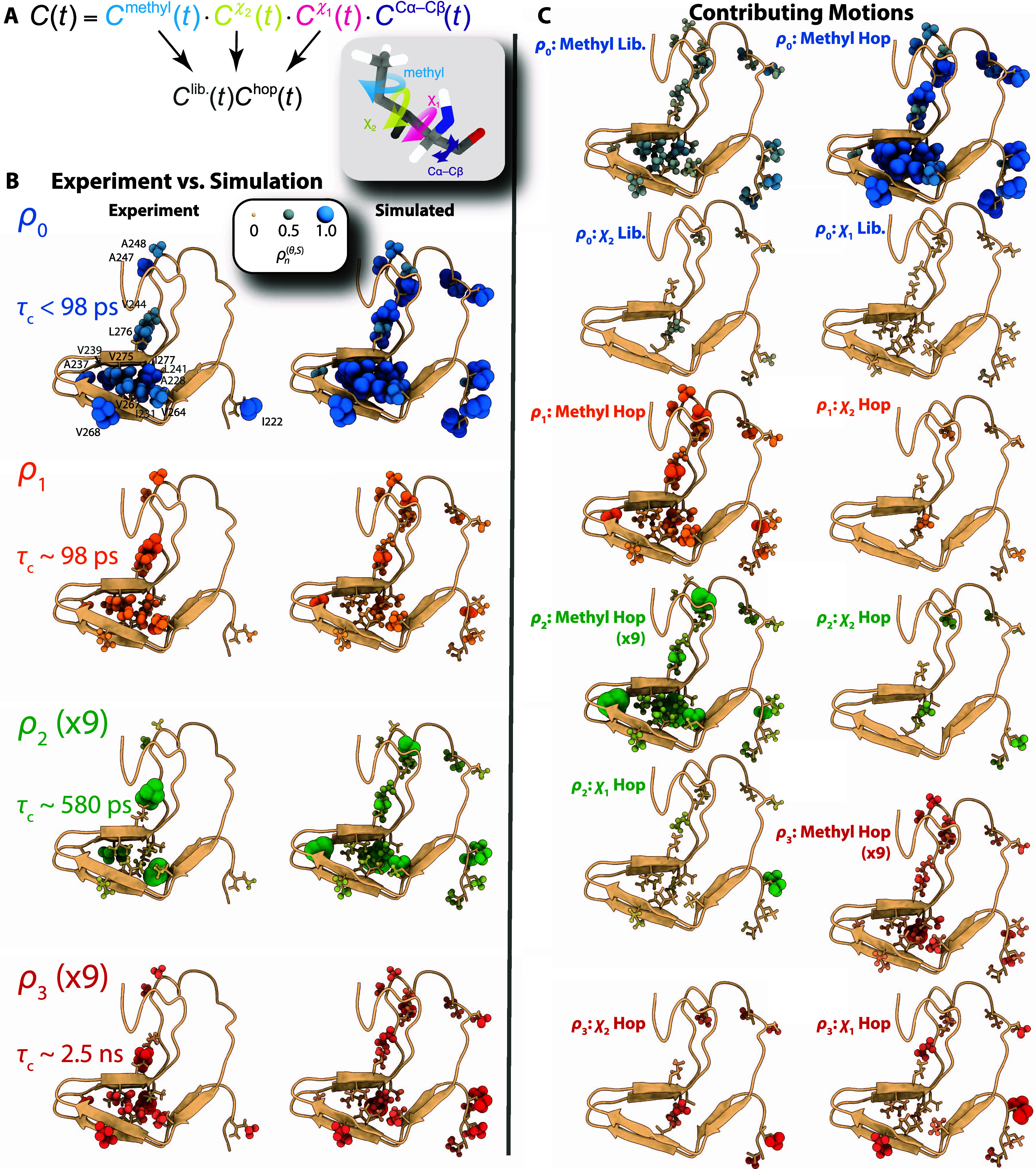
Experiment and simulation vs structure and motional decomposition. Panel (A) illustrates the decomposition of isoleucine methyl motion into 3 bond rotations (methyl: Cγ–Cδ, χ_2_: Cβ–Cγ, χ_1_: Cα–Cβ) in addition to the reorientation of the Cα–Cβ bond. Each of the 3 rotations can further be subdivided into librational and 3-site hopping motion, resulting in a product of up to 7 correlation functions for Ile and Leu (5 for Val, 3 for Ala). Panel (B) plots the experimental (left) and simulated (right) detector responses. For experimental data, side chains are only shown where we obtain the experimental data. Residues are labeled for the experimental ρ_0_ (black labels: upper layer, gray labels: lower layer). Panel (C) plots a detector analysis of the motionally separated correlation functions (MD) for each detector window. Only motions making significant contributions are shown (bar plots of data are shown in SI Figures S8 and S9, and additional three-dimensional (3D) plots are shown in SI Figures S10–S13). Note that side chains are shown only where the given motion is defined for that side chain, e.g., χ_2_ hops are only defined for isoleucine/leucine, so in plots of χ_2_ hopping, alanine and valine side chains are not shown.

Methyl-bearing side chains undergo relaxation due to a variety of motions, including librational dynamics, methyl rotation, and rotameric (χ_1_/χ_2_) hopping. While not easily separable based on experiment, we have recently developed the ROMANCE approach (reorientational dynamics in MD analyzed for NMR correlation function disentanglement), which allows separation of the total correlation function obtained from MD into parts, such that
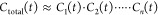
4where each of the *C*_*n*_(*t*) may be analyzed separately.^37^ Separation is achieved via a series of reference frames defined on the molecule; well-chosen frames are required for successful separation (frame validation: see SI Figure S7). In this study, we have chosen frames to separate the H–C motion of the methyl groups into methyl rotation, χ_1_ rotation (Val, Leu, Ile), χ_2_ rotation (Leu, Ile), and reorientation of the Cα–Cβ bond, as illustrated in Figure 2A for Ile. Each rotation is furthermore separated into discrete 3-site tetrahedral hops and smaller amplitude libration, for a total of 7 separate motions for Ile/Leu, 5 for Val, and 3 for Ala.

Figure 2B plots the experimental (left) and simulated (right) detector analyses for the best MD simulation onto the HET-s structure, with color intensity and atomic radii encoding the detector amplitude (ρ_0_(*z*) is redefined in MD to exclude motion slower than 3 ns). Figure 2C then shows the separation of this motion into up to 7 components. For each detector, only frames making a significant contribution are shown (see SI Figures S8–S13 for all plots). Note that due to the product in eq 4, faster motions scale the influence of slower motions in the total correlation function. This is especially important for methyl motion, for which the equilibrium value of the correlation function is 1/9, which then reduces the influence of χ_1_/χ_2_ hopping by the same factor. Then, when plotting detector responses for ρ_2_/ρ_3_ and χ_1_/χ_2_ hopping and methyl hopping, we multiply the methyl hopping by 9 (since it does not scale itself) in order to have these motions on the same effective scale as the χ_1_/χ_2_ hopping. It is less clear if ρ_1_ should be scaled since there is some overlap in the time scales of the motions.

From Figure 2C, we learn, first, that ρ_0_ (<98 ps) is determined almost entirely by librational motion and methyl hopping. ρ_1_ is dominated by methyl hopping, whereas ρ_2_ and ρ_3_ have significant contributions from χ_1_ and χ_2_ motion. Cα–Cβ motion makes almost no significant contributions to any detector, except weak contributions at I219 (see SI Figure S13). We may also determine what motions are not well reproduced by MD. For example, ρ_2_ for L241 is significantly overestimated by MD (Figure 2B); an examination of Figure 2C suggests that MD underestimates the methyl hopping rate, resulting in a large contribution in methyl hopping for ρ_2_ that should instead be found in the range of ρ_0_/ρ_1_.

### Parameterization of Methyl Dynamics

**Figure 3:**
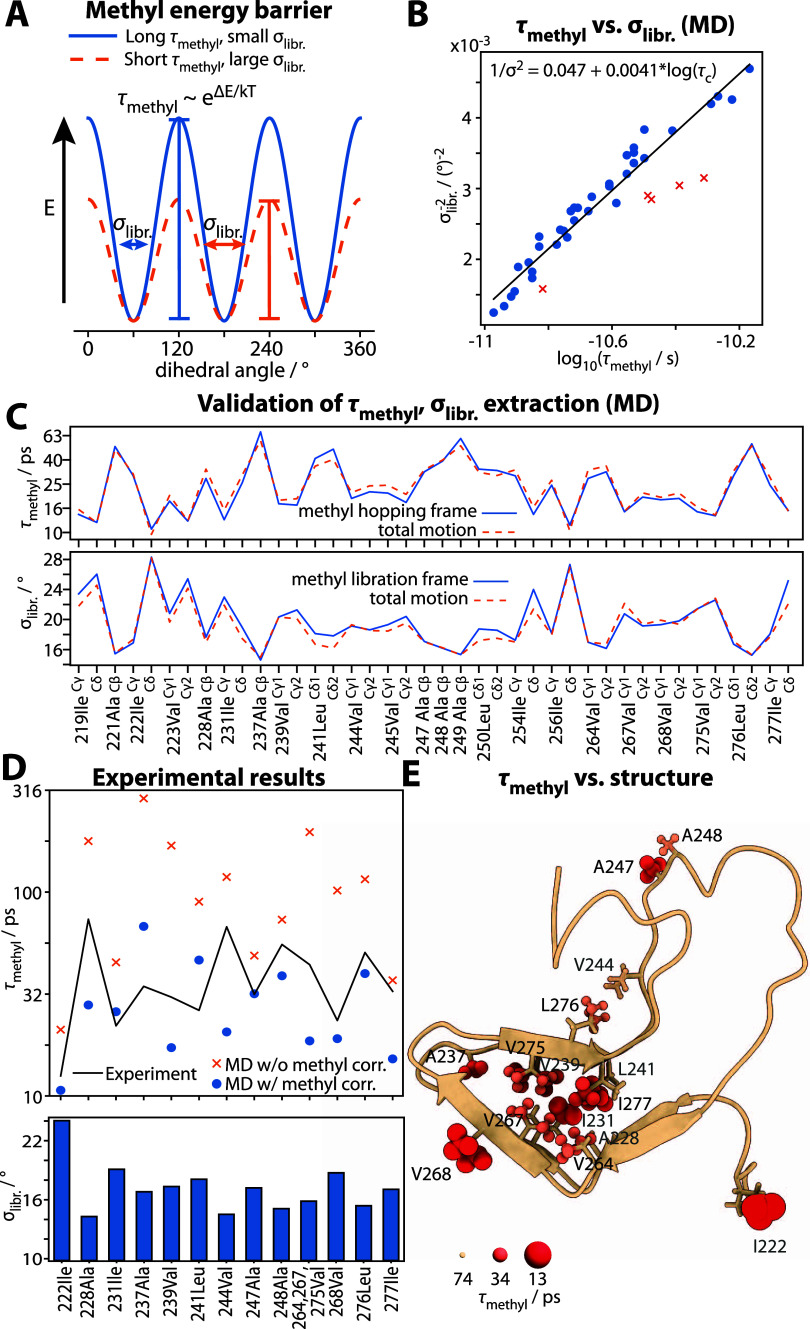
Modeling of methyl hopping and libration. Panel (A) illustrates the dihedral energy of a methyl group, where reducing the energy results in faster methyl hopping and a larger librational motion. Panel (B) plots log_10_(τ_methyl_) vs (σ_libr._)^−2^ extracted from MD data and the corresponding linear fit. Five data points are excluded for falling outside 2σ of the initial fit (red crosses). Panel (C) validates the proposed fitting approach to extract the methyl rotation rate and librational amplitude from MD data. Solid blue lines show τ_methyl_ (top) and σ_libr._ (bottom) extracted from the corresponding frames, whereas dashed orange lines show the same parameters extracted from the total motion. Panel (D) shows the results of applying the same fitting procedure to experimental data, yielding both τ_methyl_ and σ_libr._. τ_methyl_ is compared to values extracted from MD simulations with 4-point water and without methyl correction (orange crosses) and with methyl correction (blue circles). Panel (E) plots the experimental methyl correlation times onto HET-s(218–289), where large red atoms correspond to short correlation times and small tan atoms correspond to long correlation times.

Our force-field screening (Figure 1D) has significantly improved the agreement between experiment and simulation, although motional reproduction is nonetheless far from perfect (Figure 1C). Still, we can use the MD results to support the interpretation of experimental data (the complexity of dynamics in this study inhibits explicit parametrization as is sometimes possible^30,38^). For example, the mean correlation time of methyl hopping, ⟨τ_met_⟩ (later, we will see that this correlation time is time-dependent; thus, we refer to its mean), is the dominant contributor to ρ_0_ and ρ_1_ (Figure 2C, top). However, a non-negligible contribution from methyl libration prevents us from easily extracting ⟨τ_met_⟩. The librational amplitude and methyl correlation time should be related since both depend on the energy barrier to methyl rotation;^9,39^ i.e., as the methyl rotation barrier is decreased, methyl hopping rates and librational amplitudes should increase (Figure 3A). We determine that log_10_(⟨τ_met_⟩/*s*) vs σ_libr._^–2^ should be approximately linear (see SI Section S9.1); based on the ROMANCE analysis, it is straightforward to separate these motions and compare the relevant parameters, found in Figure 3B, where a linear relationship is indeed identified (a few methyl groups are excluded that deviate by more than 2σ from the linear fit, shown as a red x). Based on this relationship, we can extract ⟨τ_met_⟩ and σ_libr._ from the total detector responses in MD without using ROMANCE analysis. This is verified in Figure 3C, where results from extracting these parameters using ROMANCE (blue, solid) are compared to the results from extracting the parameters from the total motion (orange, dashed). Therefore, we may apply the same procedure to the experimental detector analysis, allowing us to determine ⟨τ_met_⟩ and σ_libr_ based on the experiment alone, with the results shown in Figure 3D (black line). The experimental ⟨τ_met_⟩ is also compared to values extracted from MD simulations (4-point water, with and without methyl barrier correction) and is plotted onto the HET-s amyloid structure in Figure 3E. Thus, our results demonstrate that the use of the methyl barrier correction yields better agreement of ⟨τ_met_⟩ with NMR-based experimental data, and we demonstrate how to separate influences of methyl libration and hopping.

### χ_1_/χ_2_ Rotameric Dynamics

**Figure 4:**
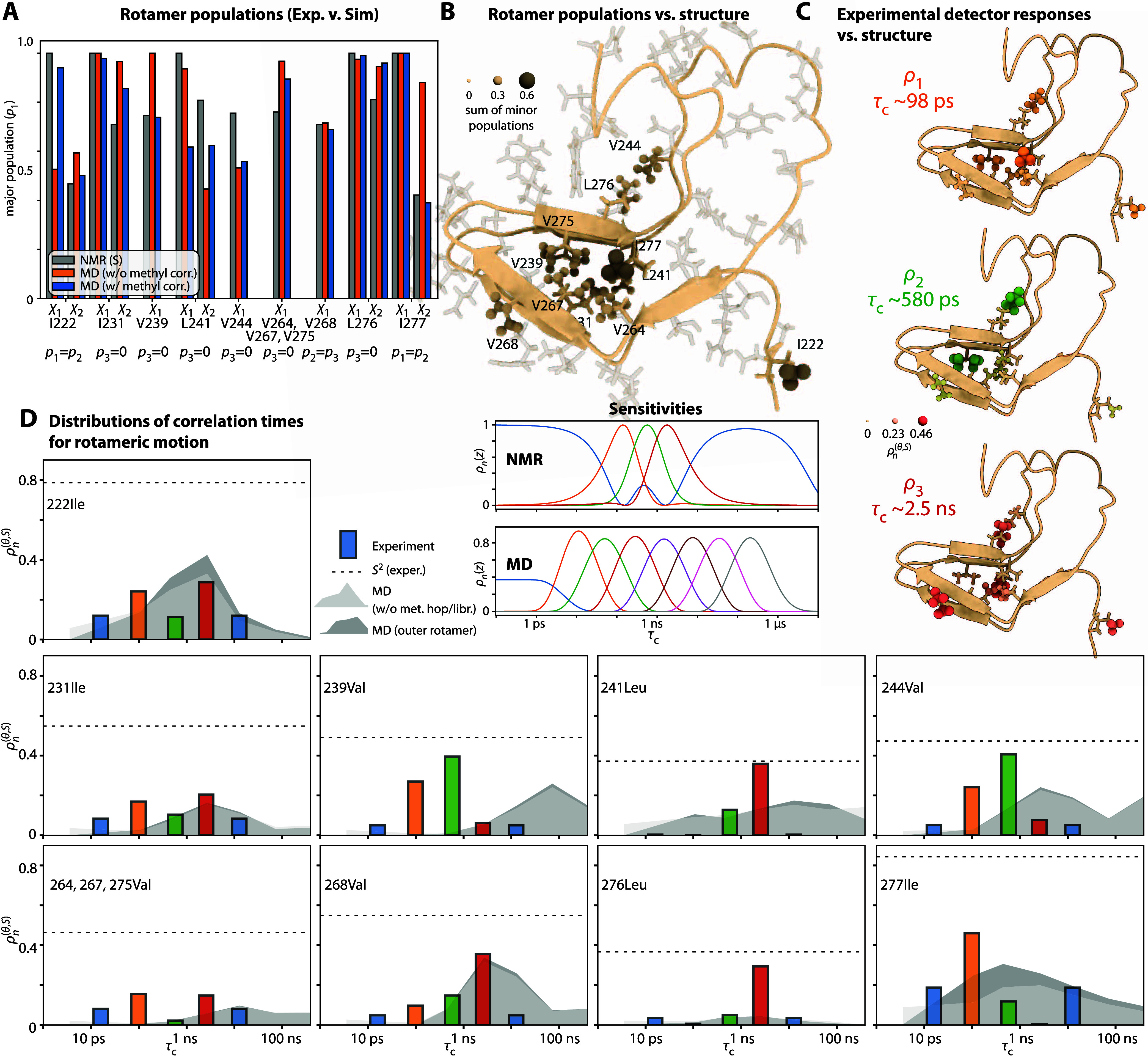
Rotamer dynamics. Panel (A) shows the major population (*p*_1_) of the χ_1_ (Cα–Cβ) and the χ_2_ (Cβ–Cγ) rotamers (*p*_1_ ≥ *p*_2_ ≥ *p*_3_, *p*_1_ + *p*_2_ + *p*_3_ = 1), as extracted from experiment and simulation. Simulations use 4-point water without (orange) and with (blue) methyl correction. For valine, only χ_1_ is defined. Experimental populations are extracted from |*S*|, assuming for isoleucine and leucine, that only one χ_1_ rotameric state is populated (*p*_1_ = 1). We select the population model based on |*S*| and based on MD results (see SI Section S10). Panel (B) plots the sum of the minor populations, 1 – *p*_1_, onto HET-s(218–289), such that larger atoms correspond to higher flexibility. Panel (C) plots detector responses ρ_1_–ρ_3_ corresponding to nonmethyl (hopping/libration) motion onto HET-s(218–289), where larger, more intense atoms indicate larger responses in the corresponding sensitivity window. Panel (D) plots experimental detector responses (colored bars) as a function of the mean detector position and compares them to MD detector responses. The experimental ρ_0_ may have significant contributions from both motions with τ_c_ < 100 ps and τ_c_ > 2.5 ns, so this response is split between both sides (blue). MD detector responses are also plotted [MD sensitivities in (D) top middle]. Light gray regions show the contributions from all motions except methyl hopping and libration, whereas dark gray shows contributions only from the outer rotamer hopping (χ_1_ for valine, χ_2_ for leucine/isoleucine). S^2^ is shown as a grey dashed line, where methyl rotation is factored out by multiplying the experimental S^2^ by 9.

Once methyl rotation and libration have been parametrized, we can analyze the remaining motion. From C, we know that χ_1_/χ_2_ libration and Cα–Cβ motion make very small contributions to the detector responses. Furthermore, for 4-point water with methyl barrier correction, usually only the outer rotamer (χ_2_ for Ile, Leu, χ_1_ for Val) makes significant contributions. An analysis of the populations in MD confirms this, where the largest population for χ_1_ rotamers of Leu/Ile is usually nearly 1, with the exception of L241 (Figure 4A). Based on experimental DIPSHIFT measurements of the order parameter |*S*|, we can estimate the major (largest) population of the outer rotamer by assuming either {*p*_1_ ≥ *p*_2_, *p*_3_ = 0}, {*p*_1_ ≥ *p*_2_ = *p*_3_}, or {*p*_1_ = *p*_2_ ≥ *p*_3_}. The model is chosen based first on |*S*| (for a given |*S*|, only two of the models are valid) and whichever is then closest to the MD simulation (model choice makes only minor differences, SI Figure S14). Results are plotted in Figure 4A.

As with H–N backbone dynamics, correcting the methyl rotation barrier improves the estimate of the rotameric populations (Figure 4A), further highlighting the relayed effect of local dynamics on more distant motion (agreement between experimental and simulated order parameters is also improved; see SI Figure S15). We also factor out methyl hopping and libration from the experimental detector responses found in Figure 1C based on parameters in Figure 3D to obtain a characterization of the correlation times resulting from the remaining motion. We expect predominantly contributions from hopping of the outer rotamer (χ_1_ for Val, χ_2_ for Ile/Leu), as is observed in Figure 2C. The results are encoded onto the HET-s structure for ρ_1_–ρ_3_ in Figure 4C and are shown as colored bars for ρ_0_–ρ_3_ in Figure 4D, where bars are positioned at the mean correlation time for each detector. Note that it is ambiguous whether ρ_0_ represents fast (<98 ps) or slower (>2.5 ns) motion, so in Figure 4D, we split the ρ_0_ amplitude into half and show it on both sides of ρ_1_–ρ_3_. For comparison, an MD-based detector analysis is performed on the outer hopping motion (dark gray) and additionally on all motions except methyl hopping/libration (light gray). Both analyses use eight integral-normalized detectors, where this normalization scheme allows us to interpret the detector response as an estimate of the amplitude of (1 – *S*^2^)θ(*z*) at each detector’s center;^28^ accordingly, the dark gray area is a good estimate of the distribution of correlation times resulting from the outer hop, whereas the light gray area also includes other nonmethyl motions (primarily χ_1_/χ_2_ libration). The inclusion of all motion adds some faster components to this distribution and also has the effect of scaling down some of the slower contributions. The simulated detector responses indicate a broad distribution of correlation times, and indeed, the experimental detector responses are consistent with this view, although in some cases, the range of time scales differ between experiment and simulation; nonetheless, rotameric populations are in fairly good agreement. Note that the agreement of the rotameric populations indicates that the energies of stable side-chain configurations are fairly well-estimated by MD. On the other hand, transitions between those states may be multistep processes, where the activation energy in the MD simulation of any given step has the potential to distort the distribution of correlation times. We regard the overall agreement as being fairly good, although by investigating the distribution of correlation times in detail, we also reveal that there remains room for improvement.

### Origin of Correlation Time Distributions

**Figure 5:**
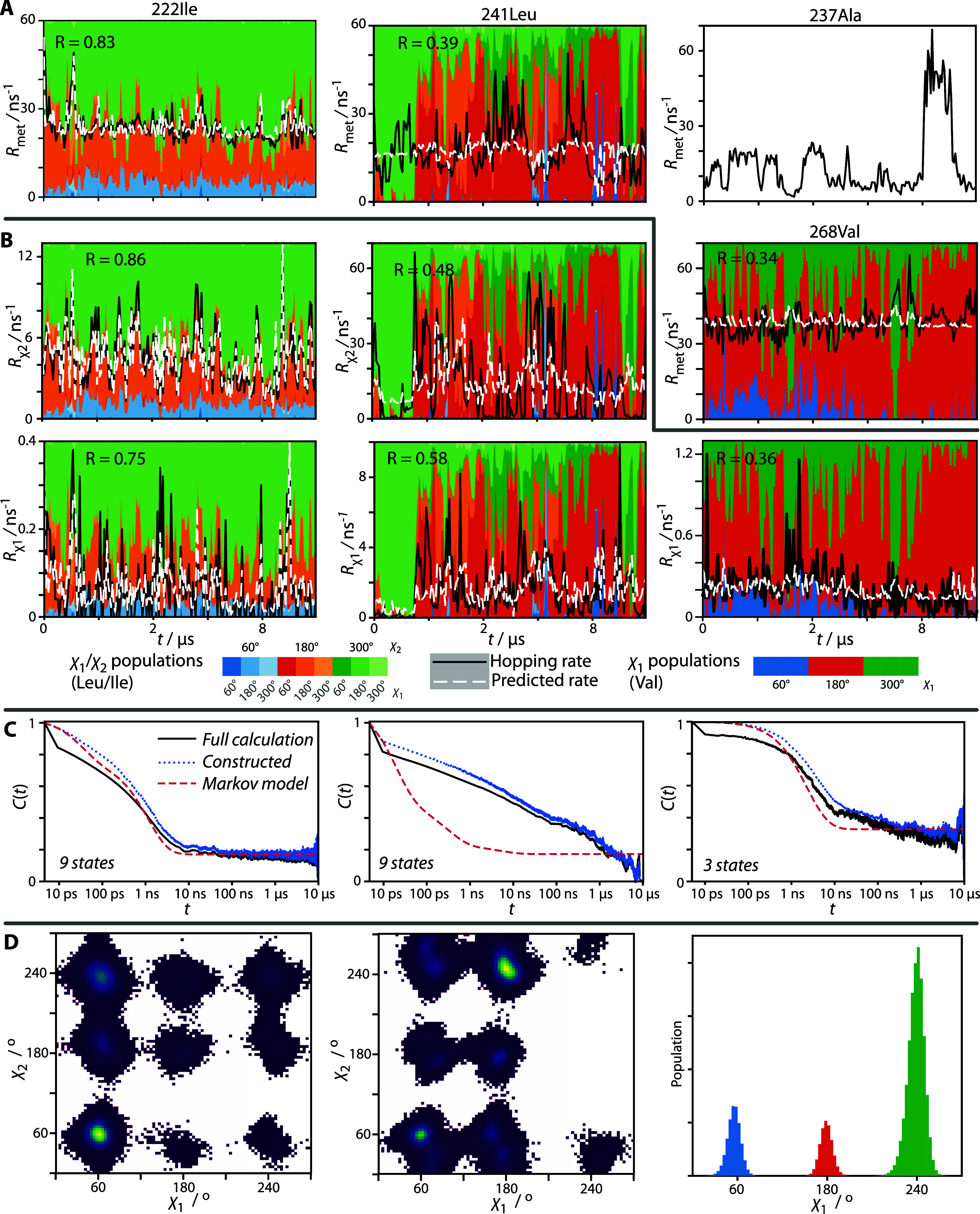
Variability of the rotamer dynamics. Panel (A) plots the mean methyl hopping rate (averaged over 50 ns bins) for four selected residues as a function of time (black, solid line), whereas panel (B) plots the χ_1_/χ_2_ of Ile and Leu or only χ_1_ for Val. In both (A/B), the background color coding indicates the fraction of the time for each bin spent with a given rotameric state (9 states for χ_1_/χ_2_ of Ile/Leu, 3 states for χ_1_ of Val, and no coding for Ala). The white dashed line indicates the predicted hopping rate based on the population of the rotameric states. Panel (C) compares direct calculation of the Cβ–Cγ (Leu/Ile) or Cα–Cβ (Val) correlation function (black, solid) with a correlation function constructed based only on the χ_1_/χ_2_ states (blue, dotted) and finally constructed based on a Markov model (magenta, dashed). Panel (D) shows the populations of χ_1_/χ_2_ as Ramachandran plots for Leu/Ile and χ_1_ as a histogram for Val. The figure is organized into columns, with *I*222 in the first column, L241 in the second column, and V268 in the third column (with A237 in the extra position at the top).

A comparison of an experiment to the simulation indicates that some residues agree significantly better than others; for example, V268 is fairly well reproduced, whereas V239 and V244 appear to have faster motion in the experiment than in the simulation. In either case, simulated results point to a broad distribution of correlation times, and experimental results are consistent with this. While it is not possible to determine the source of distributions of correlation times from our experimental data, we can investigate MD results in more detail, where we reveal a time dependence of the correlation time of hopping. To demonstrate this, we break the MD trajectory into 200 × 50 ns bins and evaluate methyl and rotameric hopping rates in each bin (simply by counting the number of hops per bin). Figure 5A shows the mean methyl hopping rate (black lines) for selected residues (SI Figures S18–S20 plots all residues), and Figure 5B plots the χ_1_ and χ_2_ hopping rates (SI Figures S21–S23 for all residues). The hopping rates indeed change over time, explaining the broad distributions of correlation times in Figure 4D rather than a narrow distribution expected for a constant hopping rate. Note that while we could fit methyl hopping data with one correlation time (Figure 3), the emergence of time dependence in Figure 5A indicates that this is indeed only an average of fluctuating methyl hopping rates.

So, what causes the time dependence? In Figure 5A/B, we show the fraction of time spent in each of 3 or 9 rotameric states for each bin (3 for Val, 9 for Ile, Leu), as indicated by the background color. In some cases, we are able to predict the bins’ hopping rates based on the time spent in each state within each bin, that is
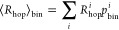
5where ⟨*R*_hop_⟩_bin_ is the average hopping rate in a bin, *R*_hop_^*i*^ is the hopping rate for the *i*th rotameric state, and *p*_bin_^*i*^ is the fraction of time spent in state *i* for the given bin. Then, the parameters *R*_hop_^*i*^ are fit to the *p*_bin_^*i*^ and ⟨*R*_hop_⟩_bin_ for all bins and used to back-calculate the hopping rate (Figure 5A/B, white, dashed lines). This works quite well for I222 but somewhat poorly for L241 (for A237, there are no rotameric states to predict the changes; the variability in methyl hopping must depend on some other factors). Indeed, I222 is found at the N-terminus of HET-s(218–289), well outside of the rigid cross-β amyloid core of the fibril, so that tight packing of neighboring side chains should have little influence. In contrast, core residues are more difficult to predict. However, note that even where correlation coefficients are low (Figure 5A/B, upper left), for periods of time in the trajectory, it is nonetheless possible to get a good estimate of hopping rates based only on populations, whereas, at other times, external factors must play a more significant role.

It then becomes possible to model the dynamics of some side chains with a Markov model,^40^ where the states of the model are the 3 or 9 rotameric states. A Markov model assumes that exchange occurs between a number of states, and the probability of moving to a given state at each time step is determined strictly by the current state occupied. To test if the various side-chain rotameric states are governed by Markov dynamics, we can calculate the correlation function for the reorientation of the Cγ–Cδ bond from MD simulation directly (Figure 5C, black lines). However, this includes librational motion, which we cannot capture with the Markov model. To eliminate this, we construct a trajectory based only on which of the 3 or 9 states the side chain is determined to occupy and recalculate the correlation function (without assuming Markov dynamics). Finally, we construct the Markov matrix, exchange matrix, and correlation function resulting from the Markov model (see SI Section S12). We see then, that I222 motion is well-described by a Markov model depending on the χ_1_/χ_2_ states, whereas L241 is not, indicating that its motion depends on other factors (i.e., hidden variables^41^), likely from its tighter packing in the fibril core. Nonetheless, we note that Markov models can be successfully applied to rotameric dynamics in other systems.^42^ Results for all residues are found in SI Figures S26 and S27.

### Side-Chain–Side-Chain and Side-Chain–Backbone Coupled Dynamics

Where hopping rates are not determined by the current state of the χ_1_/χ_2_ rotamers, we expect the surroundings to influence the hopping behavior. In the fibril core, for example, we envision some extra space allowing for one or more residues to sample multiple rotameric states. However, as a given side chain moves to a new position, this should clear additional space for a neighboring side chain to sample new configurations. Some of these new rotameric states may even trap the first side chain in a given state while allowing space for other side chains to reorient, all in all creating a complex, coupled, time-dependent rotameric dynamics, explaining in part the broad distributions in correlation times.

**Figure 6:**
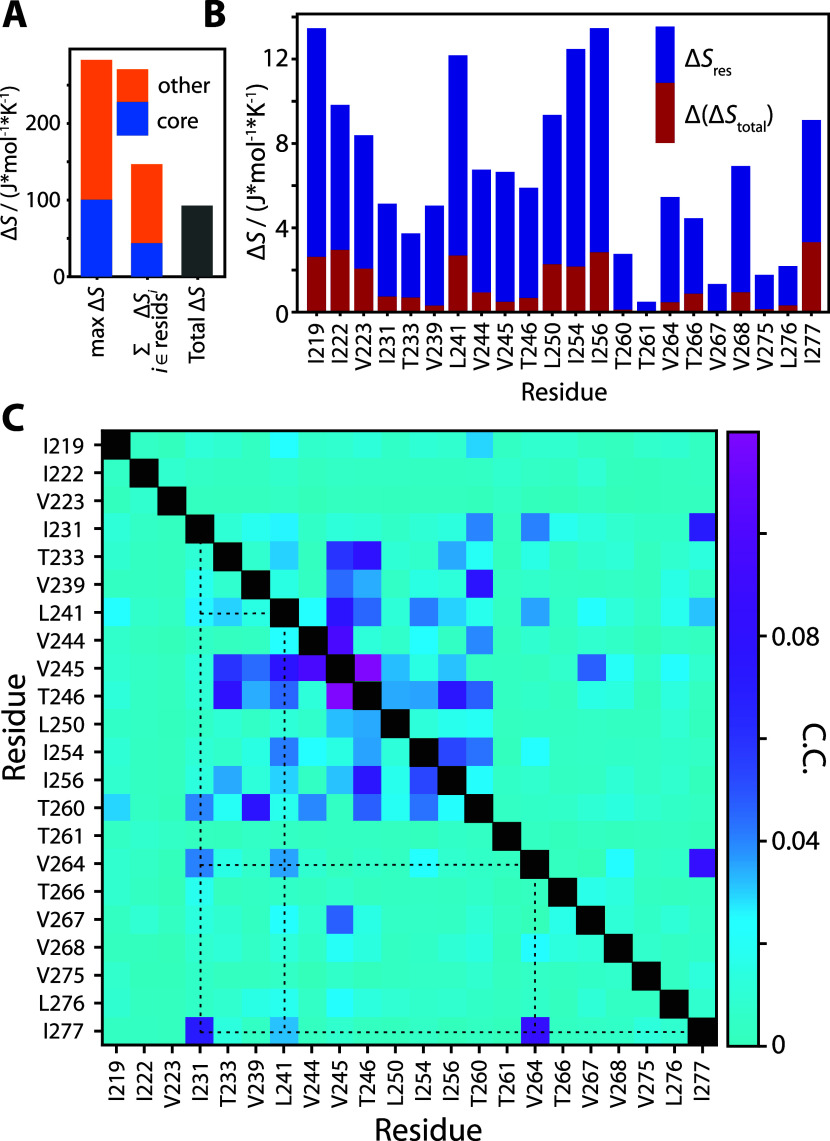
Rotameric contributions to the entropy from Ile, Leu, Val, and Thr. Panel (A) compares the total possible entropy from rotamers (i.e., equal populations for all rotameric states), the sum of the entropies from the individual side chains, and the total entropy. Panel (B) shows the entropy of each side chain (blue, Δ*S*_res_) and the change in total entropy if that side chain is not considered (red, Δ(Δ*S*_total_)). Panel (C) shows cross-correlation between side-chain rotameric states, obtained as described in the text. Diagonal elements are shown as black (c.c. = 1), and the dashed lines highlight a coupling network in the fibril core.

Such coupled dynamics should theoretically impact contributions to entropy from the side-chain rotamers. For example, the nine rotameric states for Ile/Leu should, if equally populated, yield an entropy contribution of Δ*S* = −*R*∑_*i* = 1_^9^*p*_*i*_ log *p*_*i*_ = *R* log 9 = 18.3 J/mol^–1^ K^–1^, whereas unequal populations will lead to a lower entropy (*R* is the ideal gas constant and *p*_*i*_ are the populations, here assumed to all equal 1/9). If we have two or more side chains, their entropy contributions are additive only if their motions are independent of each other. However, the complex situation described above will lead to the correlation of rotameric states and as a result will reduce the total entropy. To investigate this effect, Figure 6A first plots the total possible entropy from all rotamers of Val, Thr, Ile, and Leu as a reference value (max Δ*S*) and compares this to the sum of entropy over all individual residues. The latter calculation does not account for correlation among the rotameric states, so it will overestimate the entropy in the case of significant correlation. Finally, we calculate the total rotameric entropy, considering the configurations of all side chains simultaneously. The total Δ*S* is significantly less than the sum over all side chains, confirming a significant correlation among the rotameric states. We also determine the entropy of the individual side chains (Δ*S*_res_) and then calculate the change in the total side-chain entropy if the given residue is omitted (Δ(Δ*S*_total_)). This indicates how independent a side chain’s configurations are from its neighbors. In Figure 6B, we see that the latter calculation is typically a small fraction of the former, indicating that while a given side chain may sample a number of states for a fixed configuration of the other side chains, the configurational sampling of the side chain becomes highly restricted. Finally, we may use entropy to evaluate the correlation between pairs of residues by constructing correlation coefficients from the entropy of two residues. For residues p and q, we may calculate 2(Δ*S*_p_ + Δ*S*_q_ – Δ*S*_p,q_)/(Δ*S*_p_ + Δ*S*_q_), which yields 0 if the rotameric states are independent (Δ*S*_p_ + Δ*S*_q_ = Δ*S*_p,q_), but 1 if they are fully dependent (Δ*S*_p_ = Δ*S*_q_ = Δ*S*_pq_). The results for all pairs of methyl-bearing residues are shown in Figure 6B. Note that the individual cross-correlations are not very large, although Figure 6B indicated that a given side chain’s possible configurations were highly restricted based on the other side chains. This indicates that variations in the side chain configurations are not dominated by pairwise interactions but rather the net effect of all nearby chains. Nonetheless, we do observe a network of correlations for some of the core residues: I231, L241, V264, and I277 (dashed lines). Interestingly, L241 and I277 also move in general more independently from their neighbors than most other residues in β-sheets. More flexible residues near and in the loop region (244–260) also exhibit a strong correlation, in contrast to flexible residues near the N-terminus (I219, *I*222, and V223).

**Figure 7:**
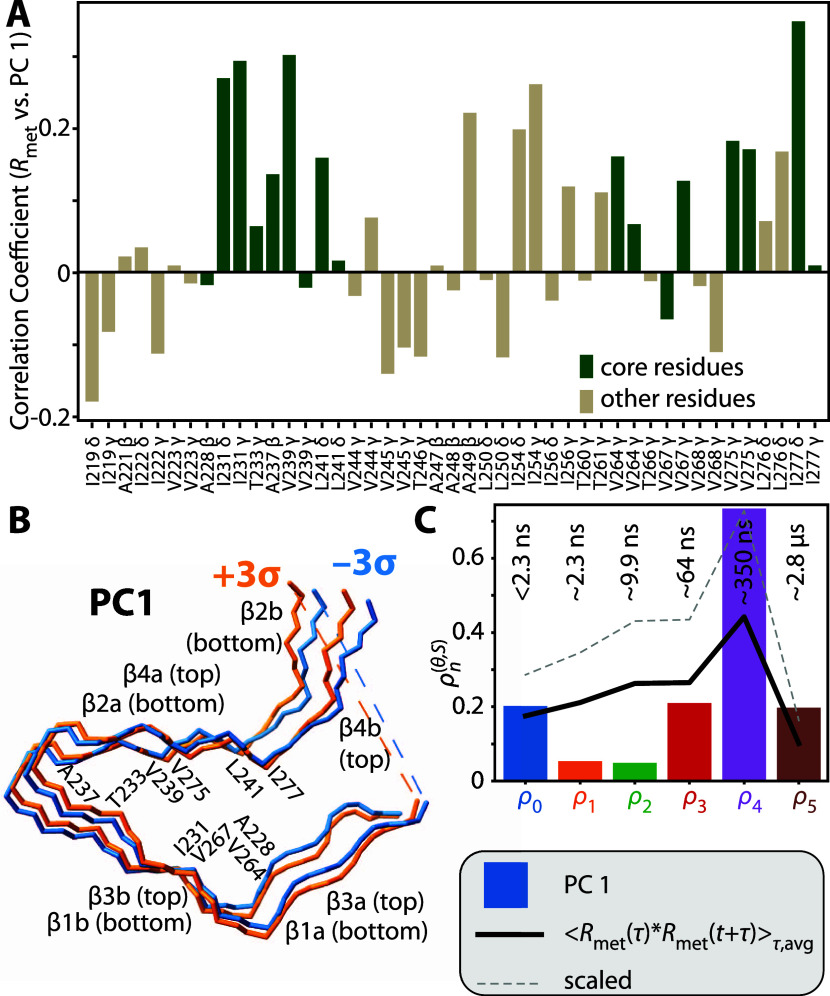
Correlation of methyl rotation rates with PC 1 motion. Panel (A) plots correlation coefficients of PC 1 with the methyl rotation as a function of residue, with core residues highlighted in green. Panel (B) shows the deviation of the average backbone HET-s structure due to PC 1 motion (± 3σ shown). Panel (C) shows detector analyses of the time-correlation function for PC 1 (bars) and the averaged methyl relaxation rates for core residues (black). Both correlation functions have been normalized such that *C*(0) = 1, *C*(∞) = 0. A gray, dashed line scales the detector analysis for methyl relaxation rates such that it matches the detector analysis of PC 1 for ρ_4_.

A final potential source of fluctuating dynamics is the variation of the total space in the fibril core. A few recent studies have investigated the role of breathing in determining the hopping of aromatic rings in proteins observed via NMR,^11,25,43^ including in HET-s(218–289) and related HELLF fibrils.^44^ Breathing motion refers to the concerted expansion or contraction of the protein. Breathing can be described by one or more motional modes, which have a net effect of increasing space in the protein core (these modes should be governed by Poisson statistics rather than having a regular frequency). These modes must always be present, but the questions are: what is the time scale of their motion, and are their amplitudes large enough to allow processes such as ring flipping? In the case of HELLF amyloid fibrils, aromatic side chains in the fibril core did not undergo ring flips, indicating that these breathing motions did not have sufficient amplitude. Here, we investigate breathing via principal component analysis (PCA),^45^ where we determine the largest 10 principal components of the HET-s(218–289) β-sheet regions (N, C’, Cα in residues 225–245, 261–281), and find the time dependence of the principal components. From this, we may calculate correlation coefficients between the principal components and the methyl hopping rates. The full set of results is found in SI Figures S29 and S30; while the resulting correlation coefficients never exceed 0.45 for any side chains, we find that the methyl hopping rates of all but 3 core methyl groups are positively correlated with PC 1, shown in Figure 7A (core side chains shown in green). Figure 7B plots the deviation from the mean HET-s(218–289) backbone structure due to PC 1 (± 3σ), where we see a slight opening of the triangular β-solenoid fold at β1a/β3a vs β2b/β4b. Given its small size, it is not surprising that this motion does not vastly affect internal dynamics. Some residues may not exhibit a positive correlation due to the local structure. For example, A228 is a small side chain in a region with more space, so it may not be affected by fibril compression. V239 and V267 fall in a region where the backbone position does not vary much for PC 1. L241 and I277 also exhibit almost no correlation, but from Figure 4A, we know that these are highly dynamic, implying plenty of space nearby, potentially also reducing the impact of PC1 on these residues.

Interestingly, we find that the time scale of motion of PC 1 manifests in the time dependence of the methyl hopping rates. We can observe this by calculating the time-correlation function of PC 1 and performing detector analysis on the result. This is compared to detector analysis of the normalized time-correlation function of the methyl hopping rates (*C*_*R*_met__)(*t*) = ⟨*R*_met_(τ)·*R*_met_(*t* + τ)⟩_τ_, averaged over all core residues. In Figure 7C, we see that the maximum response is found for both correlation functions near 350 ns, indicating that the methyl hopping rate has a component varying on the time scale of PC 1. However, we expect other, likely faster, local variations in structure that influence the methyl hopping rate, explaining the larger responses at shorter correlation times for the methyl hopping.

This mode dynamics, potentially including other principal components, can explain the coupling of methyl rotation rates to backbone dynamics (Figure 1D). Collective modes previously observed for the backbone^17^ should result in a minor compression of the fibril core (e.g., Figure 7B), where a decreased methyl rotation barrier may allow the core to more easily reconfigure to adapt to that compression. In other words, the core pushes back less in this case, modifying the amplitudes and correlation times of the collective backbone motion. While the coupling is apparently weak, as indicated by small correlations between backbone principal components (breathing) and methyl rotation rates (Figure 7A), the best and worst agreement between backbone experiment and simulation nonetheless vary by more than a factor of 3. More accurate rotameric populations due to methyl correction (Figure 4A) may also help improve backbone dynamic reproduction. Indeed, a major challenge of the application of MD simulations to solving complex dynamics is the subtle dependence of motion in the trajectory on many force-field and simulation parameters. However, it is of note that minor force-field improvements can have an impact on the overall dynamics.

## Conclusions

Many of the dynamic characteristics of HET-s side chains and backbone can be attributed to a tightly packed fibril core: individual core side chains are highly limited in their configurational sampling for any given configuration of neighboring chains (Figure 6B), backbone modes couple to methyl rotation where only minor compression of the fibril backbone impacts methyl rotation rates (Figure 7), most core residues populate primarily one configurational state with the exception of L241 and I277 (Figure 4A), and packing of those residues creates a coupled side-chain network with I231 and V264 (Figure 6C).

While a tight packing of hydrophobic side chains is important for fibril stability,^46^ to avoid entropically expensive interaction of water with these side chains, dynamic contributions to fibril stability must also be considered. For example, Xue et al. showed that poor-quality protein structures often exhibit a high energy barrier for methyl hopping. This is because while the van der Waals interactions of methyl groups and their surroundings may not be particularly high for these structures, if the methyl group is rotated, significant steric clashes occur.^9^ These clashes reduce the contributions of methyl motion to the entropy, making it less likely to be the correct structure. Note that decreased rates of 3-site methyl hopping do not impact a structure’s entropy unless hopping stops entirely; rather, reducing the methyl librational amplitude reduces entropy, which also depends on the methyl rotation barrier^39^ (e.g., σ_libr._ in Figure 3D is more relevant than τ_methyl_ to entropic stabilization). Furthermore, both our results and those of Xue et al. indicate the variation of the methyl rotation barrier in time, with one source of variation being protein breathing,^11^ showing that this type of global protein motion, while perhaps not having a significant direct effect, is nonetheless important to protein stability. Sampling of different rotamers in fibrils also contributes to their overall entropic stability,^12,13^ so that structural stability depends on the complex interplay of methyl and rotameric dynamics and their coupling to other motional processes. Therefore, while a tight fibril packing is important to avoid unfavorable hydrophobic interactions, this must nonetheless be balanced against maintaining stabilizing dynamic processes.

Packing of the side chains of I231, L241, V264, and I277 additionally seems to have some effect on HET-s function, where mutation of I231 or I277 to alanine reduces HET-s activity, apparently due to reduction of the hydrophobicity of the core.^47^ Interestingly, the double mutant I231A/A228 V recovers some of its lost activity by introducing a larger side chain at the 228 position so that the key feature appears to be the total space occupied by the hydrophobic side chains. I277 and L241 differ considerably from I231 in that they are both considerably more dynamic (Figure 4A), and they also exhibit more motion independent of neighboring residues (Figure 6B). I277 is more important for retaining HET-s activity than L241, where I277A mutants reduce HET-s activity, but L241A mutation has a much lower impact.^47^ Interestingly, L241 and I277 are well stacked one on top of each other along the fibril axis, with L241 and I277 on the first and second winding layer of the β-solenoid, respectively. We speculate that the increased mobility at this amino-acid position (241 for the first winding layer and 277 for the second) is important in fibril formation, where it has been shown that the C-terminal end (which includes I277) is crucial to template the elongation of incoming HET-s monomers onto the fibril structure.^48^ Note that we recently reported the atomic structure of HELLF amyloid fibrils, a very close functional homologue of HET-s,^49^ with both amyloid fibrils sharing an identical backbone structural core. I277 is replaced by glutamine in HELLF without perturbing the backbone structure, suggesting that the presence of a hydrophobic residue at this amino-acid position is not necessary to fold a rigid canonical β-solenoid fold. Here, our analysis suggests that the 241/277 positions, although enabling the hydrophobic side chain to point inside and stabilize the amyloid core, might maintain a certain level of molecular mobility to provide the required plasticity during the amyloid assembly process to template and convert a monomeric subunit into its amyloid state to elongate the fibril.

In this study, we obtain significant insight into the influence that tight packing has on the dynamics of methyl-bearing side chains. The framework introduced here, however, can also be used more generally to investigate interfaces of protein assemblies, where hydrophobic side-chain packing plays an important role in assembly formation. For example, we see via both experiment and simulation that tightly packed leucine and isoleucine residues (e.g., I231, L276) exhibit significantly less motion than those that are fully solvent-exposed (*I*222) or are near the end of a protein–protein interface (L241, I277). Valines also report on packing, where V267 and V275 indeed are tightly packed and exhibit very little motion, but V244 and V268 are solvent-exposed and are more dynamic (V223, V245, and V264 also follow this trend but are observed via MD only). V239 is an exception, occurring in the fibril core but nonetheless having similar rotameric populations as V268 (via both NMR and MD). Thus, experimental and/or simulated determination of the rotameric populations of these residues can be applied for the investigation of interaction strengths between interfaces in protein assemblies.

The techniques applied in this study may also be extended for application in other systems and side chains. For example, the methyl group in methionine may be used as a dynamics probe,^50,51^ as has been applied in Aβ_1–40_ studies,^13,14^ but has an additional bond to the methyl group. In this case, ROMANCE analysis may still be applied by simply adding additional frames to separate motion about the third rotamer (Cα-Cβ, Cβ-Sγ, and Sγ-Cδ; note that no new functionality would be required to the ROMANCE code; one just modifies arguments to existing frames). Indeed, the approaches here may also be applied to other side chains, for example, to describe ring flips in tyrosine or phenylalanine.

While fairly robust, ROMANCE analysis may not be applicable when rotations about χ_1_ and χ_2_ become correlated or if motions occur on similar time scales since ROMANCE assumes uncorrelated motions and time scale separation (ROMANCE limitations have been explored in detail in ref (37)). However, in systems lacking the tight packing in HET-s, Markov models may be applied to describe the rotameric dynamics for side chains, where exchange among all possible rotameric states is treated simultaneously.^42^ More advanced methods of defining Markov states can also help enable the application of Markov models.^52^ It should, moreover, be possible to combine ROMANCE and Markov modeling, where motions within each state of the Markov model are separated from motion due to transitions between Markov states. Time scale overlap between rotameric hopping motions may also be addressed with a Markov model approach. A final concern with ROMANCE is its performance if the time scales of hopping and libration are not well separated, which can occur if there is a low energy barrier for hopping. In this case, ROMANCE is still expected to perform well based on previous tests, indicating its robustness to time scale overlap.^37^

More challenging is the extrapolation of the simulated results in order to interpret experimental data. In general, our ability to interpret experimental motion as hops about specific bonds depends on the overall complexity of the side-chain motion. For example, we could estimate populations of the outer rotamer (χ_2_) for isoleucine and leucine in this study, but only because simulated results (and their agreement with the experiment, Figure 4A) implied that only one of the inner rotameric states was highly populated. In case both inner and outer rotamers have multiple states populated, separating the rotameric motions would not be possible based on relaxation of the ^13^Cδ alone (rotameric populations have been estimated based on chemical shift;^53^ rotameric dynamics could also be separated based on a combination of DIPSHIFT data for ^13^Cγ and ^13^Cδ for isoleucine).

Based on our framework, we are able to disentangle a number of factors influencing side-chain dynamics. Particularly interesting is that minor adjustments to the force field have wide-ranging effects, here resulting in improved rotameric populations and backbone dynamics. While this suggests a bright future for studies that quantitatively compare experimental and simulated parameters, it also leaves an open question as to what extent results from experimentally unverified MD simulations can be interpreted. Our view is that simulation provides powerful insights into determining what types of motions can be present and what factors influence those motions, but that high-quality experimental data remains indispensable to ultimately verify that dynamics in MD are relevant to the real system. While we must keep limitations in mind, we nonetheless expect that this approach can also be used as a general tool to investigate interfaces in protein assemblies, where side-chain dynamics are directly impacted by packing at the interface. Such an approach will be powerful going forward in order to identify important interactions in biomolecular assemblies and extract highly detailed descriptions of side-chain motion.

## Data Availability

All Python code used to analyze MD and experimental data is available via Github (https://github.com/alsinmr/HETs_Methyl_archive) and has been permanently archived on Zenodo (https://zenodo.org/doi/10.5281/zenodo.10104072)

## Supporting Information Available

Additional details on sample preparation, NMR experiments, fitting with INFOS,^54^ setup of MD simulations, and additional figures showing data for all ILVA residues in HET-s (PDF)

## Abbreviations

NMR: nuclear magnetic resonance
MD: molecular dynamics
NOE: nuclear Overhauser effect
CSA: chemical shift anisotropy
ROMANCE: reorientational dynamics analyzed in MD for NMR correlation function disentanglement
