# Supplementary material for: Analytical Framework to Understand the Origins of Methyl Side-Chain Dynamics in Protein Assemblies
Source: J Am Chem Soc. 2024 Mar 13;146(12):8164–78. doi: 10.1021/jacs.3c12620 (PMC10979401; doi:10.1021/jacs.3c12620)
Supplement: Supplementary file 1 — ja3c12620_si_001.pdf [file ja3c12620_si_001.pdf]

Supporting information for:

An Analytical Framework to Understand the Origins of Methyl Sidechain Dynamics in Protein Assemblies

Kai Zumpfe,[1] Mélanie Berbon,[2] Birgit Habenstein,[2] Antoine Loquet,[2]* Albert A. Smith[1]*

[1]*Leipzig University, Institute for Medical Physics and Biophysics, Härtelstraße 16-18, 04107, Leipzig, Germany*

[2]*University of Bordeaux, CNRS, Bordeaux INP, CBMN, UMR 5248, IECB, Pessac, France*

albert.smith-penzel@medizin.uni-leipzig.de

a.loquet@iecb.u-bordeaux.fr

Table of Contents

1. HET-s sample preparation 2

2. Experiments and Parameters 3

3. NMR spectrum assignment and fit 4

4. Experimental relaxation rate constants and fits 6

5. MD Simulation 8

6. S² for tetrahedral hopping 9

7. Backbone dynamics vs. force fields 10

8. ROMANCE decomposition 10

9. Methyl dynamics 16

9.1 Librational amplitude vs. methyl correlation time 16

9.2 Order parameter as a function of librational amplitude 16

10. Extracting rotamer population from *S* 17

11. Methyl and Rotameric Hopping 19

12. Markov model construction and correlation function extraction 27

13. Entropy equilibration 32

14. Correlation of principle components with methyl dynamics 33

15. References 35

1. HET-s sample preparation

Culture media preparation: The composition of minimal M9 medium is 6,8g/L Na_2HPO_4 (Sigma S7907), 3g/L KH_2PO_4 (Sigma P5655), 0.5g/L NaCl, pH7.5, 1X MEM vitamine solution (Sigma, M6895), 0,1mM CaCl2 (Sigma C1016), 10µM $ZnCl_2$ (Sigma 793523), 1 µM $FeCl_3$ (Sigma 157740), 1mM $MgSO_4$ (Sigma 230391), 1g/L of $^{15}NH_4Cl$ (Sigma, 299251) and 2g/L of d7-D-glucose (CIL, DLM206210), 100 µg/mL ampicillin (Sigma, A9518). As for the culture, minimal M9 medium was lyophilized for several days and resuspended in D_2O (Cortecnet, CD5251P1000). *E.coli* strain BL21(DE3) pLysS was transformed with pET21-HET-S(218-289)-His6 vector and plated onto LB agar plates containing 100 µg/mL Ampicillin. As initial culture, 5 mL LB/Amp medium was inoculated with a single clone and incubated at 37°C under shaking for 6 hours. The appropriate volume of the initial LB culture was harvested (1000g, 15min) in order to start a second culture of 20 mL of labeled minimal M9 medium (1g/L of $^{15}NH_4Cl$ and 2g/L of d7-D-glucose) at an optical density at 600nm of 0,2. The culture was grown at 37°C, 220rpm until OD600= 0.7 to accustom bacteria to the labeled nitrogen and carbon sources. The culture was then harvested (1000g, 15min) and the pellet resuspended in a third culture of 50mL of deuterated labeled minimal M9 medium (1g/L of $^{15}NH_4Cl$ and 2g/L of d7-D-glucose). The culture was grown at 37°C overnight until OD600=2 to accustom bacteria to deuterated medium. Bacteria were then cultured at 37°C, 220 rpm, in a final volume of 500mL of deuterated labeled minimal M9 medium with a starting OD600 at 0,2. One hour prior induction (OD600=0,7-0,8), 2 vials of 2-($^{13}CHD_2$) methyl-4-(D_3)-acetolactate precursors (kit TLAM-$I^{\delta1}LV^{proS}$, $^{13}CHD_2$, NMRbio) were added to the culture, while 2 vials of 3-($^{13}CHD_2$)-2-(D)-L-alanine precursors (kit SLAM-A^{β} $^{13}CHD_2$, NMRbio) and 2 vials of 2-ketobutyric acid 4-($^{13}CHD_2$), 3,3 (D_2) sodium salt (kit TLAM-$I^{\delta1}LV^{proS}$, $^{13}CHD_2$, NMRbio) precursors were added to the culture 20 minutes prior induction. This combination of precursors leads to $^{13}CHD_2$ labeling of one methyl group in each Ile, Leu, Val, and Ala residue, referred to as ILVA labeling. Expression was then induced with 0.75 mM IPTG in D2O at 33°C for 20 hours, 180 rpm. Cells were harvested by centrifugation (7000 g, 30 min, 4°C) and frozen (-80°C) until purification.

Frozen cells pellets were thawed and lysed by sonication on ice in 25 mL buffer A (Tris 50 mM pH 8, 150 mM NaCl). The cell suspension was centrifuged (15 000 g ,1 h, 4°C) to collect inclusion bodies. The pellet was then resuspended in 15 mL of extraction buffer B (50 mM Tris pH 8, 0.5 M NaCl, 6M Gu-HCl). The suspension was incubated overnight at 60°C, sonicated and ultra-centrifuged (250 000 g, 1 h, 25°C) before purification steps. Protein was purified over a 5 mL Histrap HP column (GE Healthcare), previously equilibrated with buffer B1 (50 mM Tris pH 8, 0.5 M NaCl, 20 mM imidazole, 8 M urea,). Protein was eluted with a step of 80% of buffer B2 (50 mM Tris pH 8, 0.5 M NaCl, 500 mM imidazole, 8 M urea), monitored by UV absorbance at 280 nm, on Akta

systems. Protein was further buffer exchanged into 150mM acetic acid pH 3.0 80% D_2O, over a HiPrep 26/10 desalting column (GE Healthcare) and pure fractions were analysed on 12% Tris-tricine SDS-PAGE. 12% (v/v) of Tris-HCl 3M pH 8 80% D2O was added to the pure solution of 0.1mM of HET-S (218-289) to reach pH 7,5. Self-assembly was promoted under rotation at room temperature for several days.

2. Experiments and Parameters

Relaxation of ^{13}C nuclei in sidechain methyl groups was used for the characterization of dynamics. The IVLA labeling scheme results in exactly one methyl group of each sidechain of isoleucine, leucine, valine, and alanine having a $^{13}CHD_2$ labeling scheme. All other positions are uniformly deuterated, with natural abundance carbon. In isoleucine, the terminal (δ) carbon is labeled, and in valine and leucine, one of the two terminal carbons are labeled, randomly distributed between the two positions. This labeling scheme provides a convenient spin-system for quantitative relaxation measurements, since the lack of neighboring ^{13}C quenches C–C magnetization transfers due to spin-diffusion and homonuclear nuclear Overhauser effects (NOE), and a single bonded proton yields narrow linewidths and simplifies the relaxation behavior of the multilevel system. Then, we use T_1 (400, 600, 700 MHz) and $T_{1\rho}$ relaxation (600 MHz, 5 kHz MAS, 12, 14, 19 kHz spin-lock strength), both of which are driven by reorientional motions of the ^1H–^{13}C dipole coupling, two ^2H–^{13}C dipole couplings, and the ^{13}C chemical shift anisotropy. This labeling scheme also enables us to utilize heteronuclear NOE (hetNOE) for dynamics in solid-state NMR (used at 400, 600, 700 MHz), which is driven solely by the ^1H–^{13}C dipole coupling. While hetNOE is commonly used in solution-state NMR dynamics studies, we are not aware of its application for quantitative dynamics characterization in solid-state NMR. For ^{15}N relaxation, low un-enhanced ^{15}N polarization and long T_1s have prevented NOE's application, but for sidechain dynamics based on ^{13}C relaxation, higher amplitude motion and higher un-enhanced ^{13}C polarization make it a straightforward experiment. In addition to these nine relaxation experiments, we also apply ^1H–^{13}C DIPSHIFT[1] to determine the H–C order parameter. All experiments were performed at 300 K. Backbone ^{15}N relaxation used for comparison to MD has been published previously.[2] All relaxation experiments were acquired as pseudo-2D spectra, with detection on ^{13}C. Experimental parameters are summarized in SI Table 1.

The pulse sequences of the four experiment types: T_1 (R_1), $T_{1\rho}$ ($R_{1\rho}$), steady-state heteronuclear (H–C) NOE, and DIPSHIFT, are given in SI Figure 1. These experiments are acquired as pseudo-2D experiments, where τ is varied, except for the NOE experiment, where we acquire a

steady-state enhancement, by acquire spectra with and without ^{1}H saturation (on/off). Parameters for the experiments are found in SI Table 1.

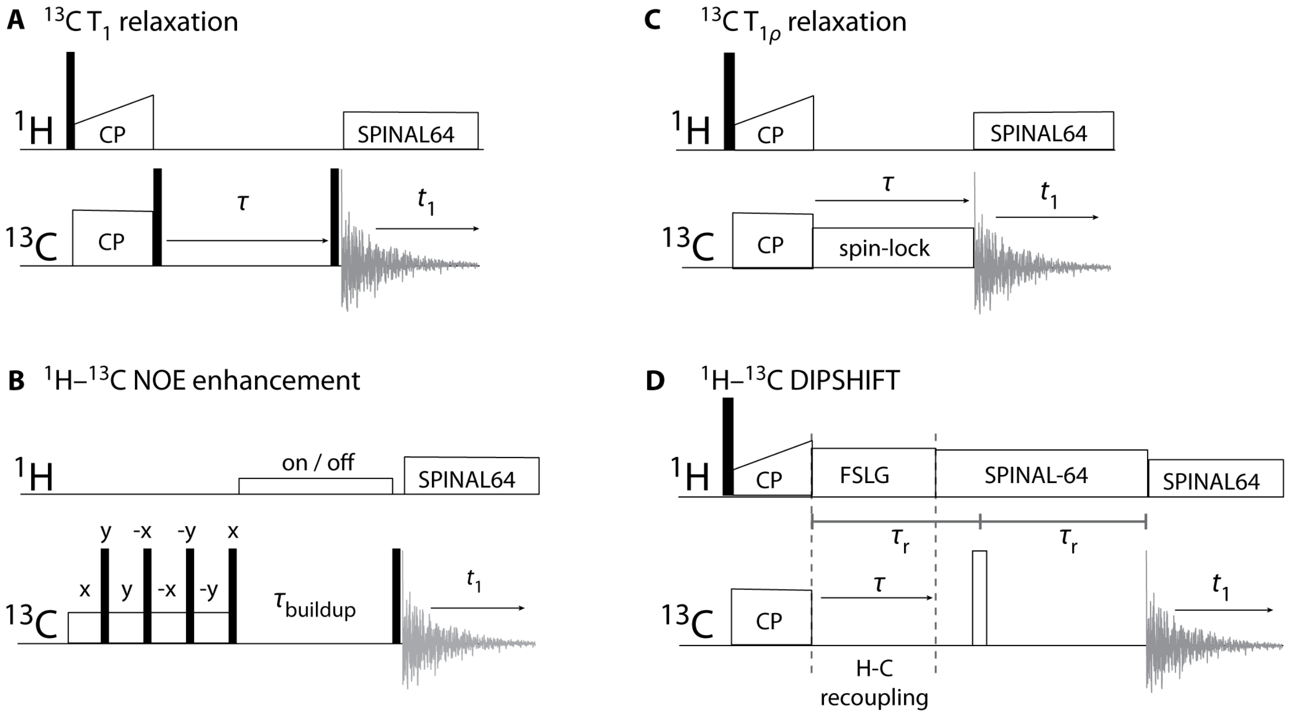

SI Figure 1. Pulse sequences. **A** ^{13}C T$_1$ relaxation with ^{1}H–^{13}C CP. The period τ is incremented to observe decay of longitudinal ^{13}C magnetization due to T$_1$ **B** ^{1}H–^{13}C NOE. Experiment is acquired twice, once with ^{1}H saturation turned on during $\tau_{buildup}$ and once with ^{1}H saturation turned off during $\tau_{buildup}$. **C** ^{13}C T$_{1\rho}$ measurement. The period τ is incremented to observe the decay of spin-locked (transverse) ^{13}C magnetization due to T$_{1\rho}$. **D** ^{1}H–^{13}C DIPSHIFT measurements. The period τ is incremented from 0 to one rotor period (τ_r) to observe evolution of ^{13}C magnetization under the ^{1}H dipole coupling.

SI Table 1: Experimental parameters

Type	Field (MHz)	Field strength (kHz)	Spinning frequency (kHz)	# Scans (# repetitions)	# Time points	Times*
R_1	400	—	5	512 (5)	19	16 ms/5.6 s
R_1	600	—	5	256 (4)	21	16 ms/11.2 s
R_1	700	—	5	1024 (9)	21	16 ms/11.2 s
hetNOE	400	—	5	1024 (5)	2 (on+off)	8 s buildup
hetNOE	600	—	5	512 (4)	2 (on+off)	12 s buildup
hetNOE	700	—	5	1024 (9)	2 (on+off)	15 s buildup
$R_{1\rho}$	600	11.6	5	512 (4)	18	0.2 ms/100 ms
$R_{1\rho}$	600	13.9	5	512 (3)	18	0.2 ms/100 ms
$R_{1\rho}$	600	18.9	5	512 (3)	16	0.2 ms/50 ms
DIPSHIFT	600	65 kHz	5	512 (4)	17	125 µs step

*The first time point for R_1 experiments is always 0, and the second to last time points are approximately log-spaced, with the second and last time points given in the table. For $R_{1\rho}$ experiments, log spacing starts from the first time point (no zero point), with the first and last time points given in the table. NOE experiments are steady-state experiments, so we only list the NOE buildup time. For DIPSHIFT experiments, time points go from 0 s to the length of the rotor period.

3. NMR spectrum assignment and fit

Experimental relaxation data was first extracted from pseudo-2D NMR spectra using the INFOS[3] package in MATLAB (https://infos.sourceforge.io). Resulting relaxation rate constants were analyzed

using the pyDR[4] package in Python (https://alsinmr.github.io/pyDR). Detector and ROMANCE analyses of MD data were also performed using pyDR. Additional analyses were also performed in Python, where scripts are available online (ZENODO ARCHIVE XX).

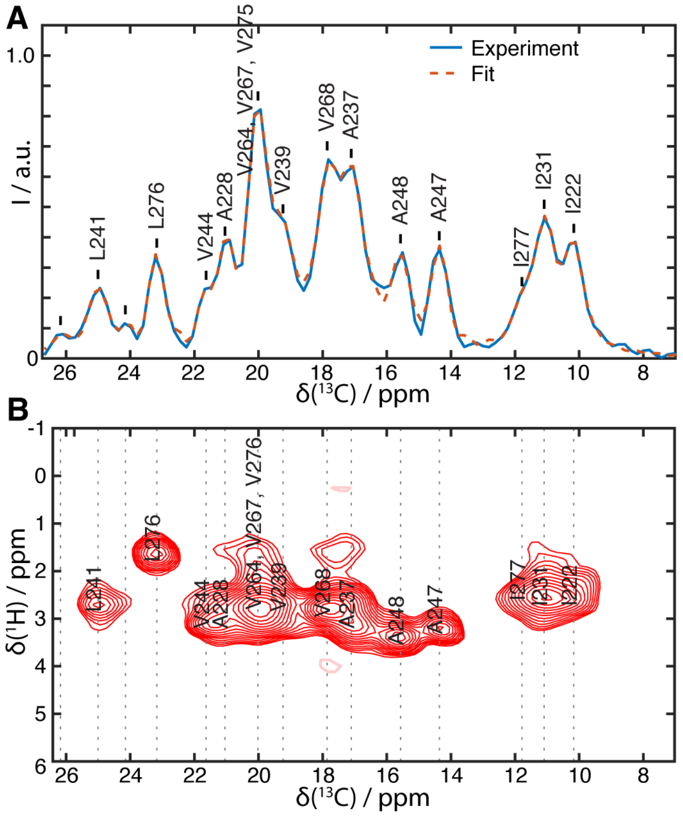

SI Figure 2. NMR 1D and 2D spectra and assignment. **A** ^{13}C 1D spectrum showing both the spectrum (solid, blue) and fit using INFOS[3] (red, dashed). Black ticks above peaks indicate the locations of fitted peaks, along with their assignment(s) above the ticks. **B** H–C correlation spectrum, with peak locations shown as dashed lines.

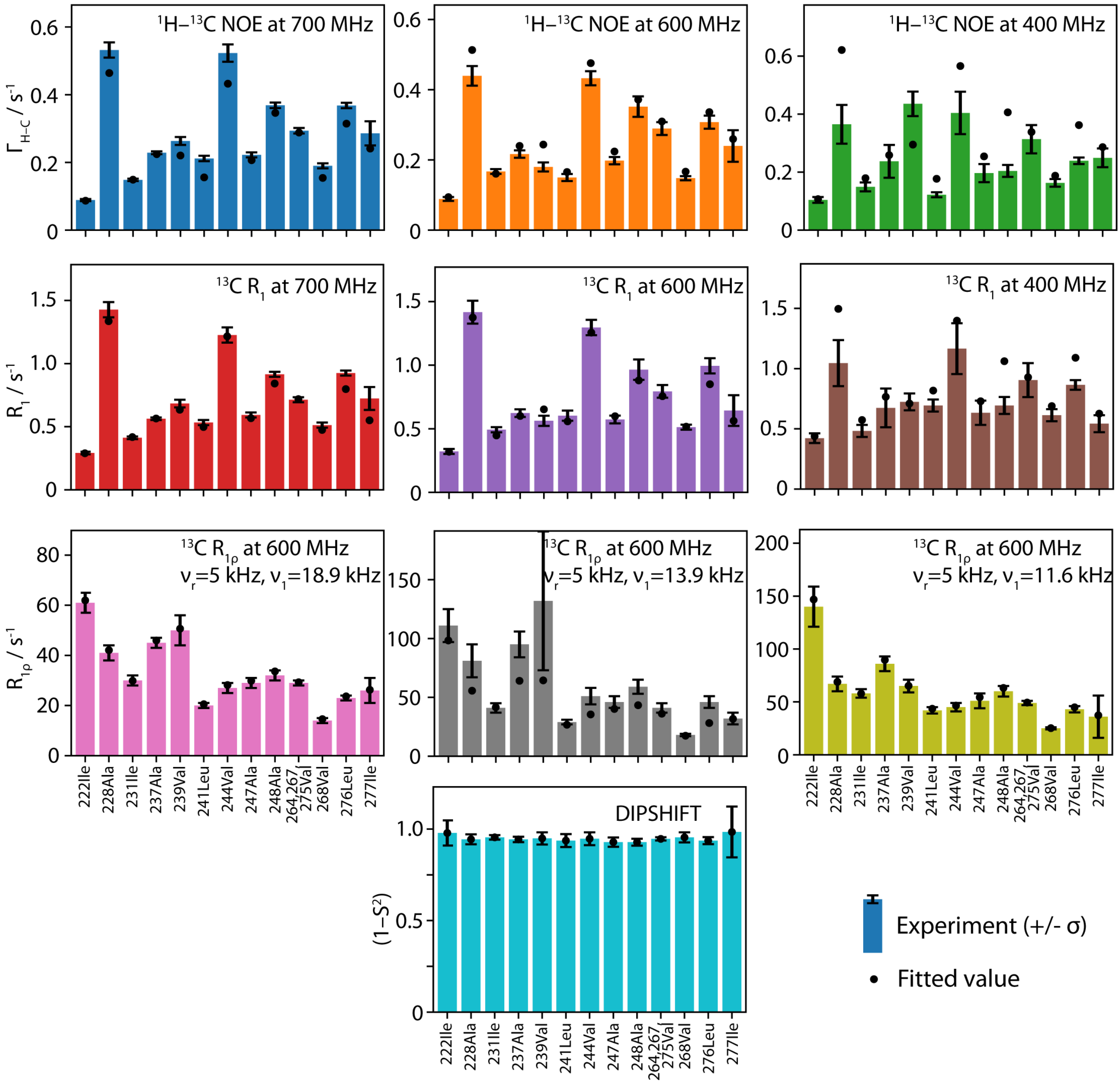

SI Figure 3. Fit of experimental data corresponding to the detector analysis in main text Figure 1C. Colored bars show the experimental value, with errorbars indicating +/- σ (determined via bootstrapping), and black circles indicate the fitted values.

4. Experimental relaxation rate constants and fits

Each series of 1D spectra, corresponding to each experiment in SI Table 1, has been fitted using the INFOS[3] software package in MATLAB. First, a reference spectrum is constructed out of the series of spectra (i.e. a weighted average of the spectra, with weighting set to optimize signal-to-noise), and that reference spectrum is fit using peak positions, linewidths, and amplitudes. From this reference, the peak positions and linewidths are fixed, and the full series of spectra is fit by varying the amplitude and the decay rate (for DIPSHIFT, we fit to simulated DIPSHIFT curves). Signal decay is modeled as a decaying exponential. For NOE spectra, the on and off signals are fit simply by varying

the amplitudes. By fitting the full series of spectra to a single parameter set, we obtain more accurate relaxation parameters. Two example fits, for R_1 and $R_{1\rho}$ data are shown in SI Figure 4 and SI Figure 5.

In order to obtain errors on the fitted relaxation rates (or dipole couplings for DIPSHIFT), we construct bootstrap data sets[5] by resampling the spectra and refitting the results. For example, for R_1 at 600 MHz, we have recorded 21 time points with 4 repetitions, resulting in 84 total spectra. Then, we randomly select 84 spectra out of that original data set (with replacement, so some of the spectra will be selected multiple times, and others will be omitted). The new "bootstrap" data set is refitted, yielding parameters that vary slightly from the original set (with variation attributed to variation between the different spectra). This procedure is repeated 96 times, and the standard deviation over the resulting relaxation parameters is calculated, and used as the uncertainties for the given experiment.

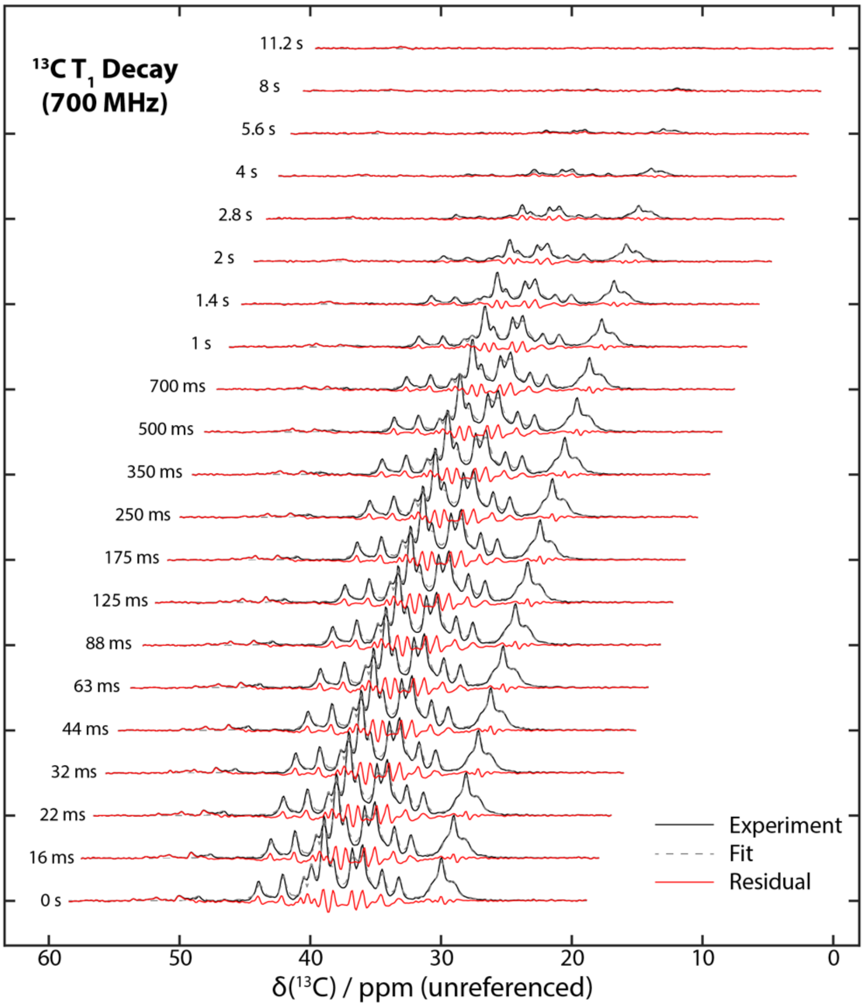

SI Figure 4. Relaxation curves and INFOS[3] fit for ^{13}C T_1 at 700 MHz. The full series of spectra is fit simultaneously, where each peak is parameterized with a chemical shift, amplitude, linewidth, and decay rate. Chemical shifts and linewidths are fit using a reference spectrum and then fixed when fitting the full series, so only amplitude and decay rate are variable in the combined fit. Each time point is shown with the experimental spectrum (black, solid), fit (grey, dashed), and fit residual (red, solid). Repetitions are averaged together in the display.

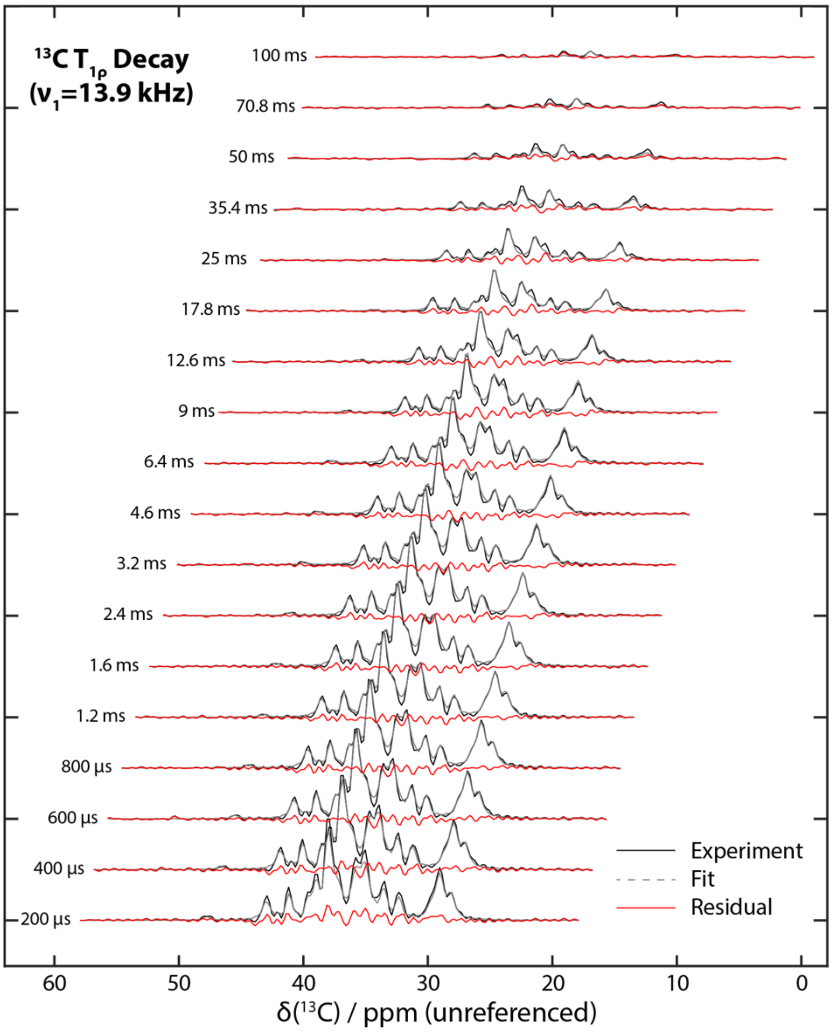

SI Figure 5. Relaxation curves and INFOS³ fit for $^{13}C\ T_{1\rho}$ for a spin-lock strength of 13.9 kHz. The full series of spectra is fit simultaneously, where each peak is parameterized with a chemical shift, amplitude, linewidth, and decay rate. Chemical shifts and linewidths are fit using a reference spectrum and then fixed when fitting the full series, so only amplitude and decay rate are variable in the combined fit. Each time point is shown with the experimental spectrum (black, solid), fit (grey, dashed), and fit residual (red, solid). Repetitions are averaged together in the display.

5. MD Simulation

All simulations have been carried out with GROMACS (version 2021.3).[6,7] For the preparation, initial structure coordinates for HET-s have been taken from PDB entry 2RNM[8] or 2KJ3.[9] For all simulations, except #5, residues 217-218 and 290-295 were removed from all three copies to produce a HET-s protein subunit 219-289.[10] The molecules have been set into a cubic box with periodic boundary conditions (PBC) and a minimum distance of 1.2 nm to the box, which was solvated with 3-point (TIP3P[11] or SPC/E[12]) or 4-point (TIP4P[11]) water and ions in form of Na^+ and Cl^- was added to a concentration of 0.15 mol (see details in table below). For all simulations, the AMBER-ff99SB-ILDN[13] force field was applied, but simulations 3-6 include a further modification of the dihedral barriers for

methyl containing amino acids.[14,15] Energy minimization was performed with the steep-integrator and a maximum number of 50000 steps. NVT and NPT equilibrium was performed every 100 ps. The final production runs were performed using the leapfrog integrator with a timestep of 2 fs at 300K. Coordinates of all atoms have been saved every 5 ps.

SI Table 2. MD simulations

Description	#H_2O	#Ions (Na^+, Cl^-)	Time	Box size
3pt-water (TIP3P, SPC/E)	14871	44	2 µs	~78 Å
4pt-water (TIP4P)	15046	44	2 µs	~78 Å
3pt-water (TIP3P, SPC/E) w/ methyl corr.	14871	44	2 µs	~78 Å
4pt water (TIP4P) w/ methyl corr.	14999	44	10 µs	~78 Å
4pt water w/ methyl corr. (2kj3)	19018	55	2 µs	~85 Å
4pt water w/ methyl corr. (5 chains)*	22733	67	2 µs	~90 Å

*only 1 nm distance to cubic box (vs. 1.2 nm for other simulations)

6. S^2 for tetrahedral hopping

In general, S^2 may be calculated if the relative orientations ($\theta_{m,n}$) and corresponding populations (p_m,p_n) of the dipole (or other anisotropic tensor) coupling are known, according to:

$$S^2 = \sum_m \sum_n p_m p_n \frac{3\cos^2\theta_{m,n} - 1}{2} \qquad \text{(S1)}$$

For 3-site tetrahedral hopping, the angle $\theta_{m,n} = \arccos(-1/3) \approx 109.5$ if $m \neq n$, and 0 otherwise. Then, the term $\left(3\,cos^2\,\theta_{m,n} - 1\right)/2$ becomes either -1/3 or 1, respectively. The equation simplifies to

$$S^2 = (p_1^1 + p_2^2 + p_3^3) - \frac{2}{3}(p_1 p_2 + p_1 p_3 + p_2 p_3) \qquad \text{(S2)}$$

For methyl hopping, the three populations must be equal, such that this becomes 1/9, corresponding to its minimum possible value for 3-site tetrahedral hopping.

7. Backbone dynamics vs. force fields

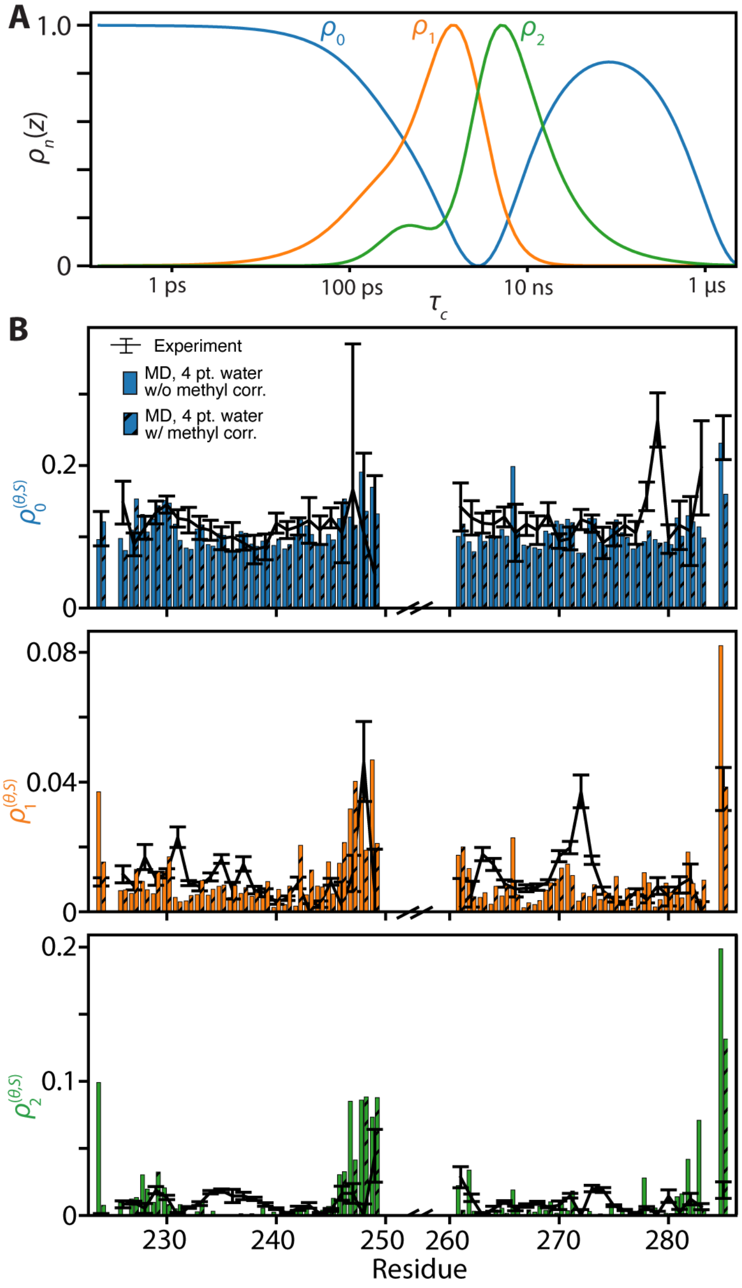

SI Figure 6. Comparison of backbone detector responses to force fields. **A** shows the detector sensitivities. **B** shows the experimental detector responses (black lines, errorbars: 1σ) vs. the detector responses from MD simulation (open bars: AMBER ff99SB-*ILDN with 4-point water, bars with '/' hatching: AMBER ff99SB-*ILDN with 4-point water and corrected methyl barrier.[14,15] Experimental backbone (H–N) data was previously published,[2] and analyzed with detectors elsewhere.[16]

8. ROMANCE decomposition

ROMANCE uses a series of reference frames to partition the total correlation function into contributions from specific motions. In the ideal case, the total correlation function of H–C motion should be equal to the product of correlation functions of the individual motions.

$$C(t) = C_1(t) \cdot C_2(t) \ldots \cdot C_N(t). \tag{1}$$

Any arbitrary set of frames can be chosen to perform this partition, but only a well-chosen set of frames, which results in statistically independent and timescale-separated motion within each set of frames, will actually yield the above equality (or at least a good approximation of it). Then, in order to validate the quality of the ROMANCE decomposition, we can calculate the product of correlation functions and perform detector analysis on both the total correlation function and the product, and compare the results, as done below in SI Figure 7. We see that the total correlation function is generally well reproduced by the product of components, thus validating the ROMANCE analysis.[17] We also plot detector analysis of the individual components of the motion onto the HET-s(218-289) molecule in SI Figure 10–SI Figure 13.

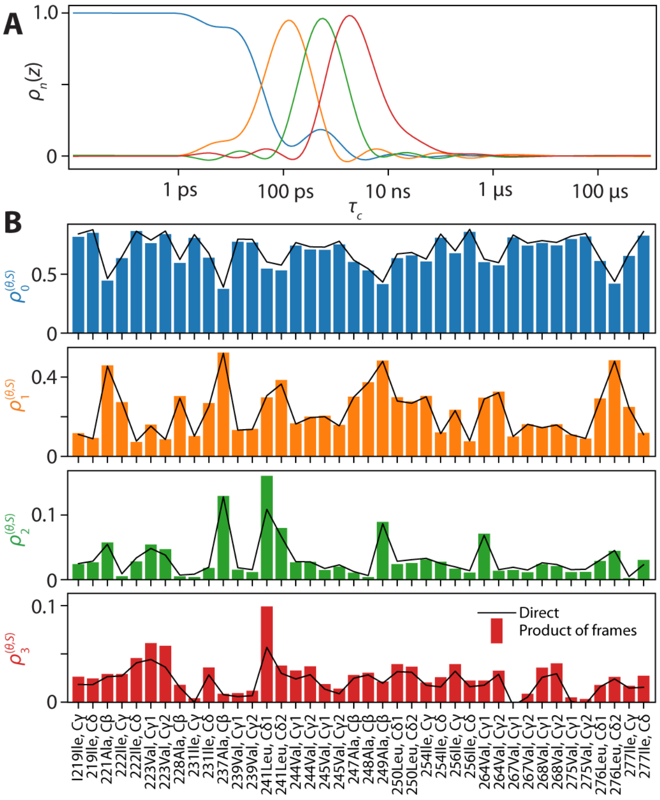

SI Figure 7. Validation of the ROMANCE decomposition (MD: 4-point water with methyl correction). **A** shows the sensitivities of the MD-derived detectors used (optimized to match experimental sensitivities). **B** compares detector analysis of the total methyl correlation functions (averaged over the three H–C bonds of each methyl group) and the detector analysis of the product of correlation functions of the individual motions.

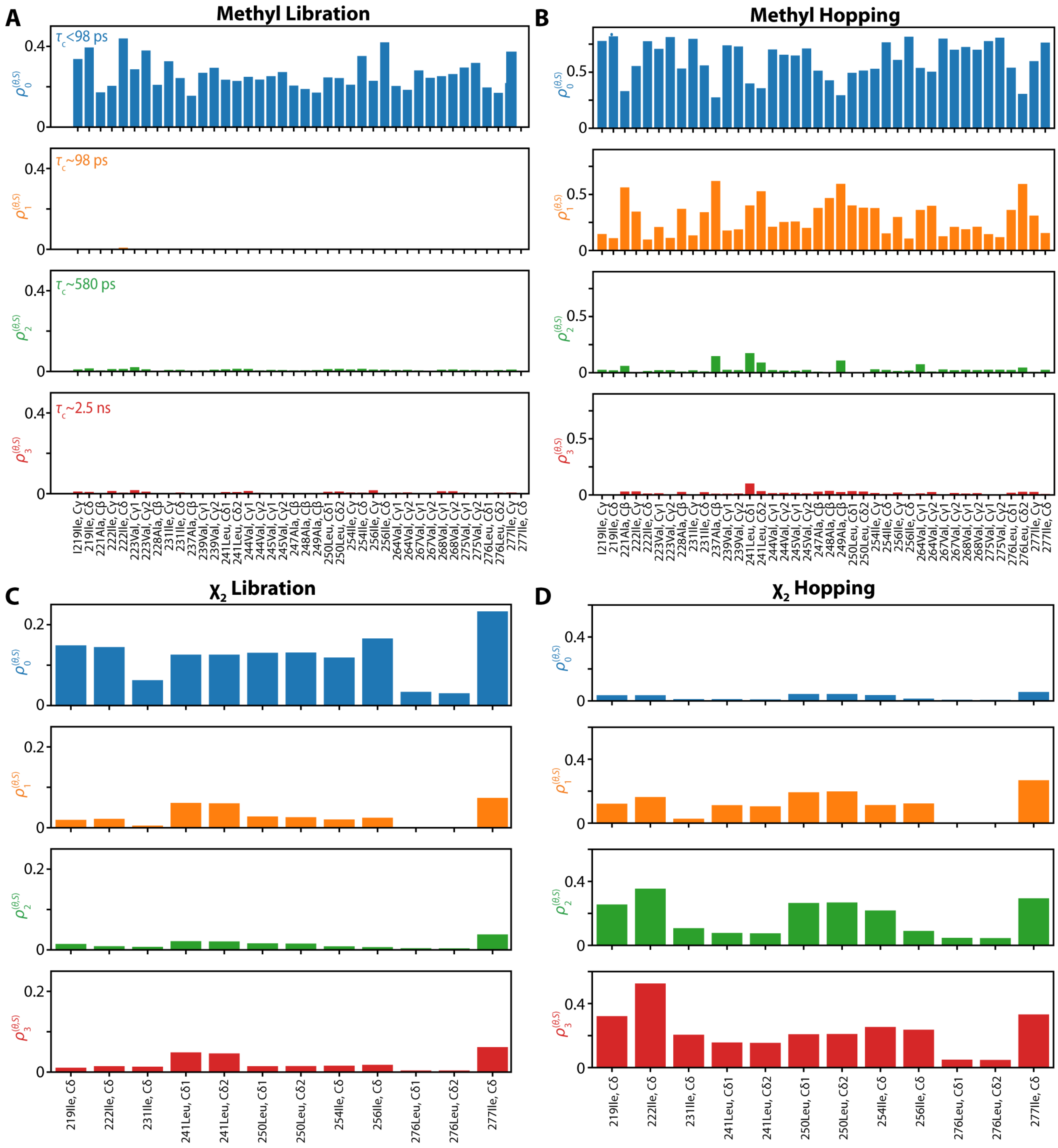

SI Figure 8. Frame decomposition of MD trajectory using 4-point water and methyl correction. **A**–**D** shows methyl libration, methyl hopping, χ_2 libration, and χ_2 hopping (other motions in SI Figure 9). Detector sensitivities are those shown in SI Figure 7A.

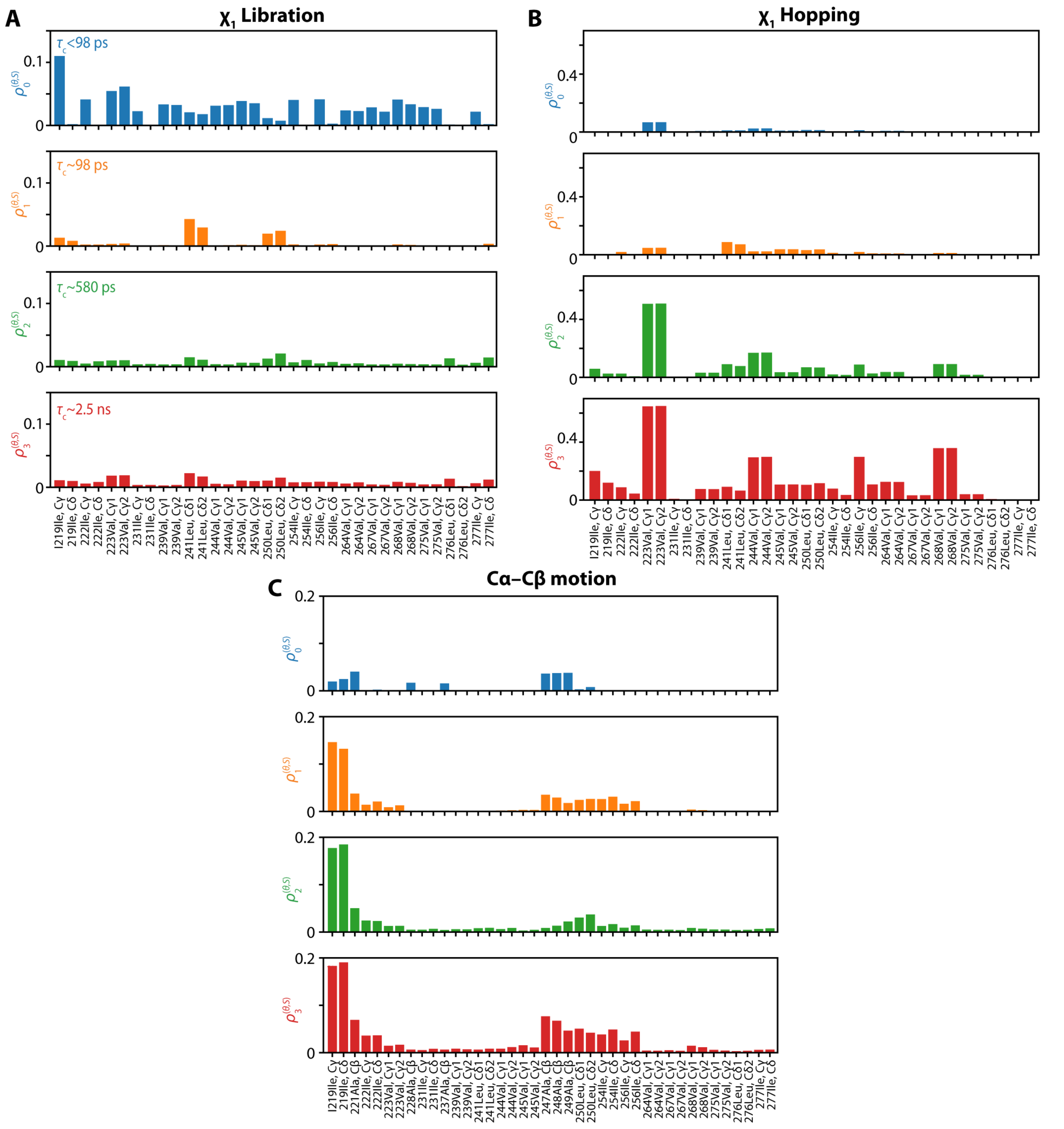

SI Figure 9. Frame decomposition of MD trajectory using 4-point water and methyl correction. **A**–**C** shows χ_1 libration, χ_1 hopping, and Cα–Cβ motion (other motions in SI Figure 8). Detector sensitivities are those shown in SI Figure 7A.

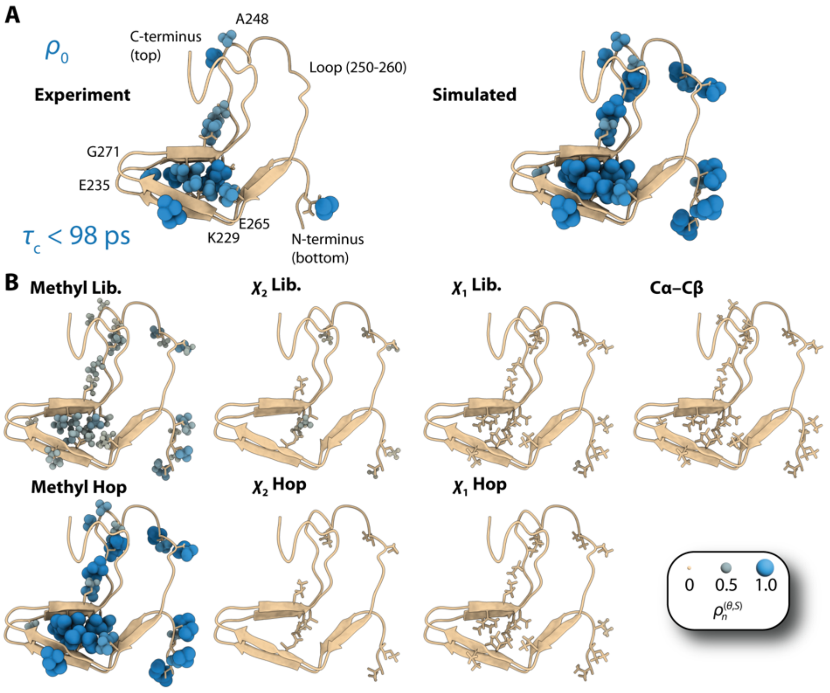

SI Figure 10. Experimental and simulated ρ_0 with frame decomposition. **A** compares the experimental and simulated detector responses (side chains only shown where we have experimental data). **B** shows the contribution of the seven motions to the simulated detector responses (side chains not shown where given motion not applicable, e.g. χ_2 for Ala, Val).

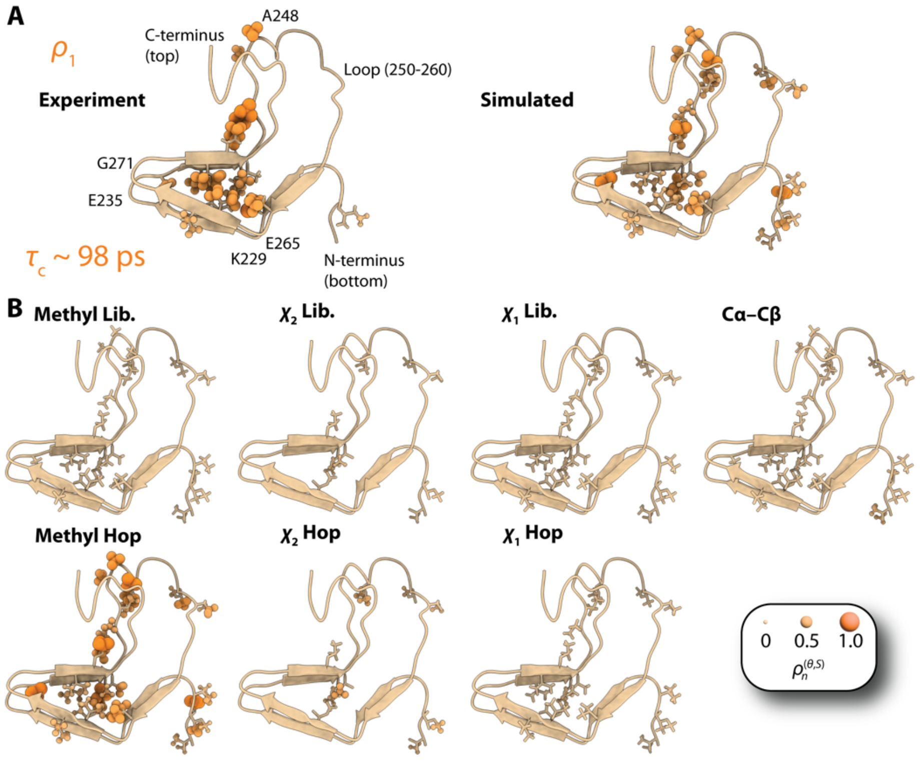

SI Figure 11. Experimental and simulated ρ_1 with frame decomposition. **A** compares the experimental and simulated detector responses (side chains only shown where we have experimental data). **B** shows the contribution of the seven motions to the simulated detector responses (side chains not shown where given motion not applicable, e.g. χ_2 for Ala, Val).

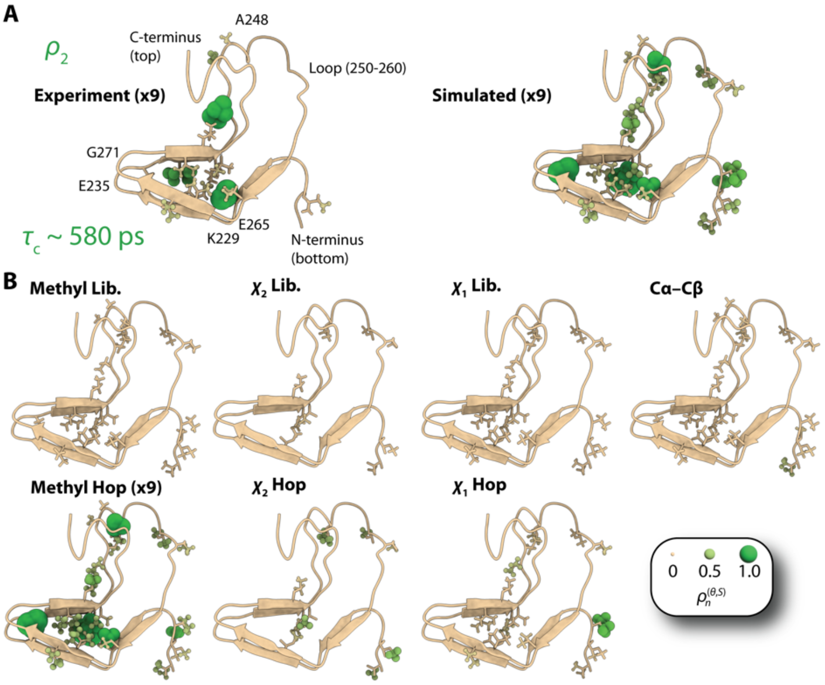

SI Figure 12. Experimental and simulated ρ_1 with frame decomposition. **A** compares the experimental and simulated detector responses (side chains only shown where we have experimental data). Responses multiplied by 9 to compensate for reduction of $C(t)$ from methyl rotation. **B** shows the contribution of the seven motions to the simulated detector responses (side chains not shown where given motion not applicable, e.g. χ_2 for Ala, Val). Methyl libration/hopping is multiplied by 9 to put it on the same scale as in **A**, whereas influence of the other motions are attenuated by the methyl hopping and so we do not scale them to match the scale in **A**.

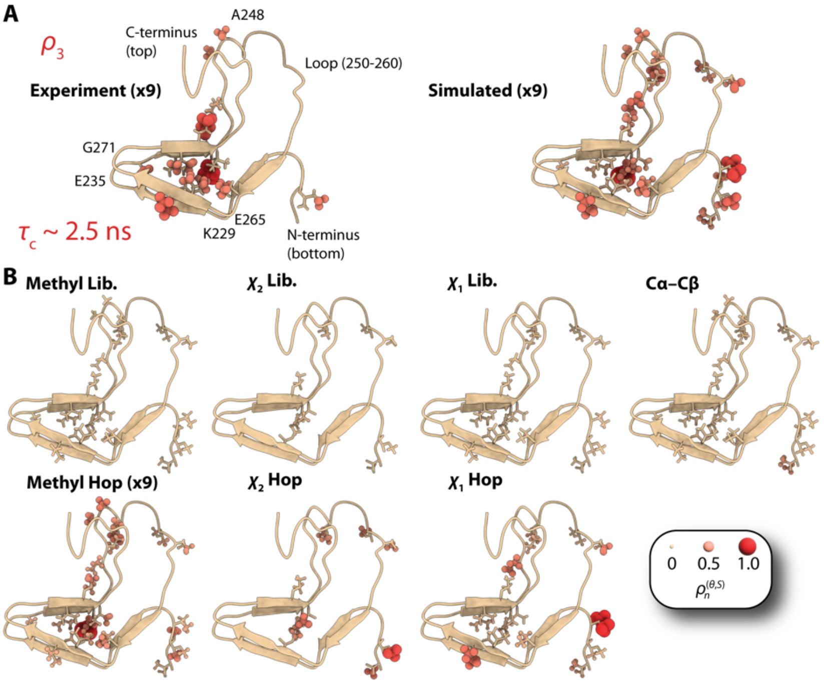

SI Figure 13. Experimental and simulated ρ_1 with frame decomposition. **A** compares the experimental and simulated detector responses (side chains only shown where we have experimental data). Responses multiplied by 9 to compensate for reduction of $C(t)$ from methyl rotation. **B** shows the contribution of the seven motions to the simulated detector responses (side chains not shown where given motion not applicable, e.g. χ_2 for Ala, Val). Methyl libration/hopping is multiplied by 9 to put it on the same scale as in **A**, whereas influence of the other motions are attenuated by the methyl hopping and so we do not scale them to match the scale in **A**.

9. Methyl dynamics

9.1 Librational amplitude vs. methyl correlation time

If we assume the potential energy for rotation of the methyl group is given by a simple cosine function, with amplitude equal to the activation energy for hopping and three energy minima, we may estimate a relationship between the librational amplitude and methyl correlation time.

$$V(\phi) = -E_a \cos(3\phi) \tag{S3}$$

Then, the correlation time of methyl hopping, according to the Arrhenius law, is simply

$$\begin{aligned} \tau_c &= \tau_{T=\infty} \exp(E_a/kT) \\ \tau_c &\propto \exp(E_a) \\ z &= \log_{10}(\tau_c) \propto E_a \end{aligned} \tag{S4}$$

We find that the log-correlation time is proportional to the activation energy. Second, if we assume for librational motion, that the probability of a given amplitude is given by its energy, then we obtain

$$p(\phi) = \frac{\exp(E_a \cos(3\phi)/kT)}{\int_0^{2\pi/3} \exp(E_a \cos(3\phi)\,/kT)\, d\phi} \tag{S5}$$

By approximating the cosine with a Taylor expansion (and dropping the offset term), this becomes a Gaussian distribution, which is consistent with the distributions of angles observed in MD

$$p(\phi) = \frac{\exp\left(-\frac{9}{2}E_a\phi^2/kT\right)}{\int_{-\infty}^{\infty} \exp\left(-\frac{9}{2}E_a\phi^2/kT\right) d\phi} \tag{S6}$$

Then, $1/\sigma^2$ for this distribution is proportional to E_a, which implies that z vs. $1/\sigma^2$ should be approximately linear.

9.2 Order parameter as a function of librational amplitude

If we assume that methyl libration is primarily a rotation of the H–C bonds around the energetic minimum, with tilt given by the tetrahedral angle ($\theta_{\text{tetra.}} = \arccos(-1/3) \approx 109.47°$), and amplitude of rotation distributed as a Gaussian, then we obtain the following expression:

$$S_{\text{libr.}}^2 = \frac{1}{2\pi\sigma_{\text{libr.}}^2} \int_{-\infty}^{\infty}\int_{-\infty}^{\infty} \exp\left(-\frac{\phi_1^2}{2\sigma_{\text{libr.}}^2}\right) \exp\left(-\frac{\phi_2^2}{2\sigma_{\text{libr.}}^2}\right) P_2\left(\vec{v}_{\phi_1} \cdot \vec{v}_{\phi_2}\right) d\phi_1 d\phi_2 \tag{S7}$$

Here, $P_2(x) = (3x^2 - 1)/2$, and $\vec{v}_{\phi_1}$, $\vec{v}_{\phi_2}$ are normalized vectors resulting from first rotation away from the z-axis by the tetrahedral angle, followed by rotation around the z-axis by the angle ϕ_α, given by

$$\vec{v}_{\phi_\alpha} = \left[\frac{2\sqrt{2}}{3} sin\,\phi_\alpha, \frac{2\sqrt{2}}{3} cos\,\phi_\alpha, \frac{1}{3}\right] \tag{S8}$$

$$\vec{v}_{\phi_1} \cdot \vec{v}_{\phi_2} = \frac{1}{9} + \frac{8}{9}(sin\,\phi_1\,sin\,\phi_2 + cos\,\phi_1\,cos\,\phi_2)$$
$$= \frac{1}{9} + \frac{8}{9}\cos(\phi_1 - \phi_2)$$

Plugging this in, we obtain:

$$S^2_{\mathrm{libr.}} = \frac{1}{2\pi\sigma^2_{\mathrm{libr.}}} \int_{-\infty}^{\infty}\int_{-\infty}^{\infty} d\phi_1 d\phi_2 exp\left(-\frac{\phi_1^2}{2\sigma^2_{\mathrm{libr.}}}\right) exp\left(-\frac{\phi_2^2}{2\sigma^2_{\mathrm{libr.}}}\right) \times \left[\frac{1}{54}(1 + 16\,cos(\phi_1 - \phi_2) + 64\,cos^2(\phi_1 - \phi_2)) - \frac{1}{2}\right] \quad \text{(S9)}$$

We define $\Phi = \phi_1 - \phi_2$, noting that the difference of these terms is also Gaussian distributed, with standard deviation of $\sqrt{2}\sigma_{\mathrm{lib}}$.

$$S^2_{\mathrm{libr.}} = \frac{1}{2\sigma^2_{\mathrm{libr.}}\sqrt{2\pi}} \int_{-\infty}^{\infty} d\Phi\, exp\left(-\frac{\Phi^2}{4\sigma^2_{\mathrm{libr.}}}\right)\left[\frac{1}{27}(8\,cos\,\Phi + 32\,cos^2\,\Phi - 13)\right] \quad \text{(S10)}$$

If we expand the cosines in a Taylor series, and truncate all powers greater than Φ^4, we obtain

$$S^2_{\mathrm{libr.}} \approx \frac{1}{2\sigma^2_{\mathrm{libr.}}\sqrt{2\pi}} \int_{-\infty}^{\infty} d\Phi\, exp\left(-\frac{\Phi^2}{4\sigma^2_{\mathrm{libr.}}}\right)\left[1 - \frac{36}{27}\Phi^2 + \frac{11}{27}\Phi^4\right] \quad \text{(S11)}$$

Since the integral is normalized, we just need to know the 0th, 2nd, and 4th moments of a Gaussian distribution with $\sigma = \sqrt{2}\sigma_{\mathrm{libr.}}$, which are 1, $2\sigma_{\mathrm{libr.}^2}$, and $12\sigma^4_{\mathrm{libr.}}$. Then, we arrive at

$$S^2_{\mathrm{libr.}} \approx 1 - \frac{72}{27}\sigma^2_{\mathrm{libr.}} - \frac{44}{9}\sigma^4_{\mathrm{libr.}} \quad \text{(S12)}$$

This formula is valid (within 5%) for $\sigma_{\mathrm{libr.}} < 24°$, after which higher order terms are required for an accurate estimate (note that this formula is only valid in radians). It is also possible to numerically integrate eq. (S10), which we have done for this study.

10.Extracting rotamer population from *S*

If we have *S*, measured experimentally (here via DIPSHIFT), and know that *S* is determined primarily by methyl rotation (hopping+libration) plus sampling of one rotameric population (e.g. just χ_1 or just χ_2), then it is possible to extract information about populations of rotameric states from *S*. Based on the order parameter, *S*, it is not possible to determine all three rotameric populations, but we may estimate the populations by comparing the results from a few different assumptions. We take the populations to be p_1, p_2, and p_3, where we define $p_1 \geq p_2 \geq p_3$.

1) Assume $p_3 = 0$, which corresponds to the magenta curve in SI Figure 14A. Since $p_1 \geq p_2$, this model is only valid for $p_1 \geq 0.5$.

2) Assume $p_2 = p_3$, which corresponds to the blue curve in SI Figure 14A. Since $p_1 \geq p_2 \geq p_3$, the smallest that p_1 can become is 1/3.
3) Assume $p_1 = p_2$, which corresponds to the orange curve in SI Figure 14A. $p_1 + p_2$ cannot exceed 1, so p_1 cannot exceed 1/2, but also may not become smaller than 1/3.

SI Figure 14A is produced by sweeping through the allowed values of p_1 under the above three assumptions, and calculating the averaged tensor at each value. Using tools provided in pyDR,[4] we return the anisotropy (δ) for the averaged tensor, and calculate $S = \delta/\delta_{\text{static}}$. For a given value of $|S|$, the uncertainty over the major population is represented by the grey region in SI Figure 14A. In SI Figure 14B, we take the experimentally determined values for $|S|$, and back-calculate the major populations based on the assumptions above. Note that for any given value of $|S|$, only two of the three assumptions are possible. Then, this plot indicates the population uncertainty for each residue due to choice of assumptions, where we see that uncertainty is quite low except for 222Ile and 277Ile.

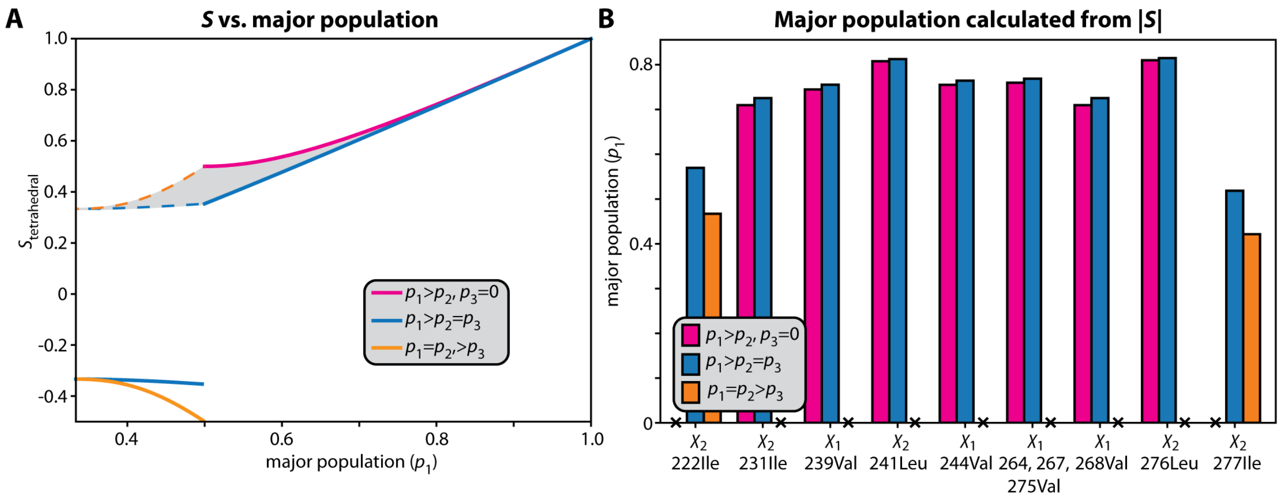

SI Figure 14. Extracting the major population from $|S|$. **A** shows S as a function of the most populated state assuming p_3=0, assuming p_2=p_3 (the two least populated states have equal populations), and assuming p_1=p_2 (the two most populated states have equal populations). Dashed lines show the absolute value of S for negative regions. For a given value of $|S|$, the major population must fall within the grey region between the extremes. **B** plots the major population as extracted from experimental values of $|S|$, using each of the three models. Note that only two of the three models can explain any given value of $|S|$, where the missing value is marked with a cross.

Note that the experimental and simulated order parameters may also be directly compared, as shown in SI Figure 15, which also confirms that the equilibrium rotamer populations are better when using the correction of the methyl rotation barrier for most residues.

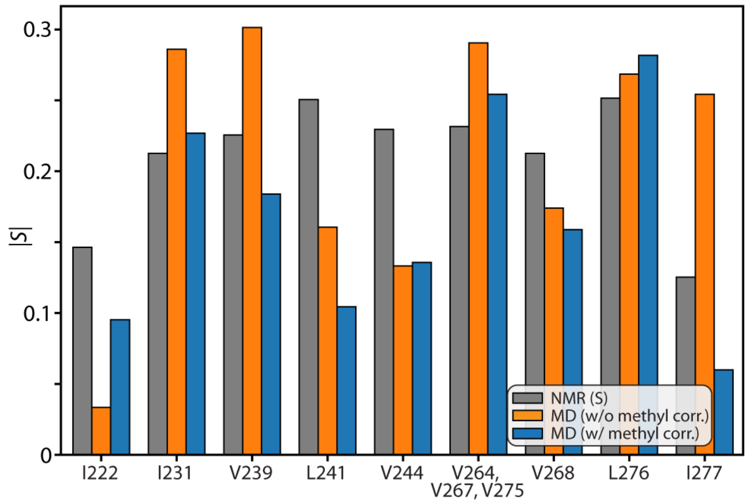

SI Figure 15. Experimental vs. simulated order parameters. Grey bars show the experimentally determined order parameters (absolute values) for each residue, whereas orange and blue bars show the order parameters calculated from MD simulation using 4-point water without and with methyl correction, respectively.

11.Methyl and Rotameric Hopping

The following section plots Ramachandran plots (Leu/Ile) and histograms (Val). For each process, we first calculate the angle of the given bond (SI Figure 16, SI Figure 17). Then, we assign each angle to a given state (0,1,2), depending if the angle is nearest to 60°, 180°, or 300°. A hop, then, is simply defined as when the state changes. Hop rates are then assessed based on the number of hops per each of 200 x 50 ns bins ($R_{\text{hop}} = n_{\text{hops}}/50\ \text{ns}$). These are plotted as a function of time in the trajectory, along with the population of each of the possible 0 (Ala), 3 (Val), or 9 (Leu, Ile) rotameric states in SI Figure 18–SI Figure 23.

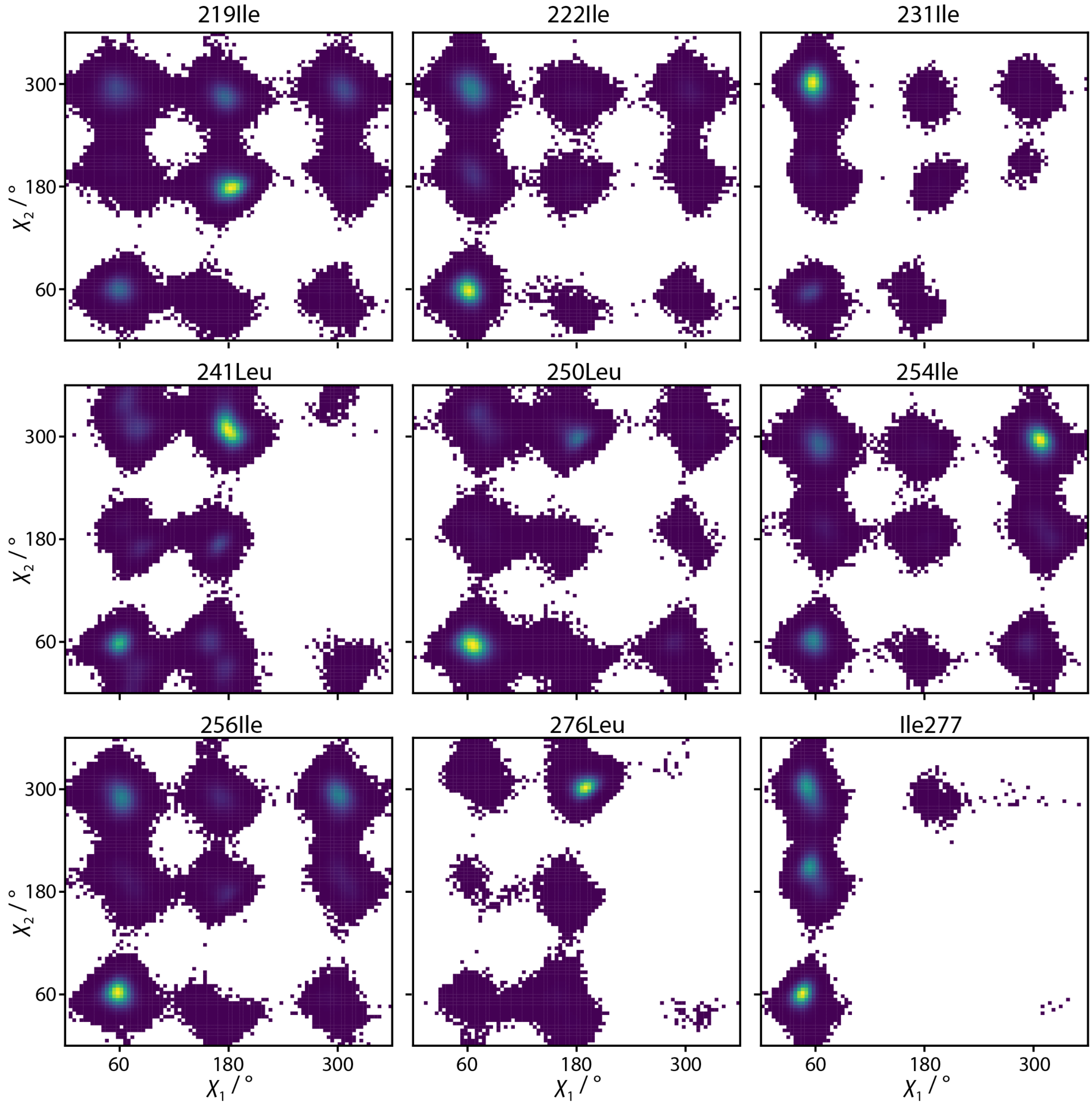

SI Figure 16. Ramachandran plots for χ_1 and χ_2 angles of leucine and isoleucine residues.

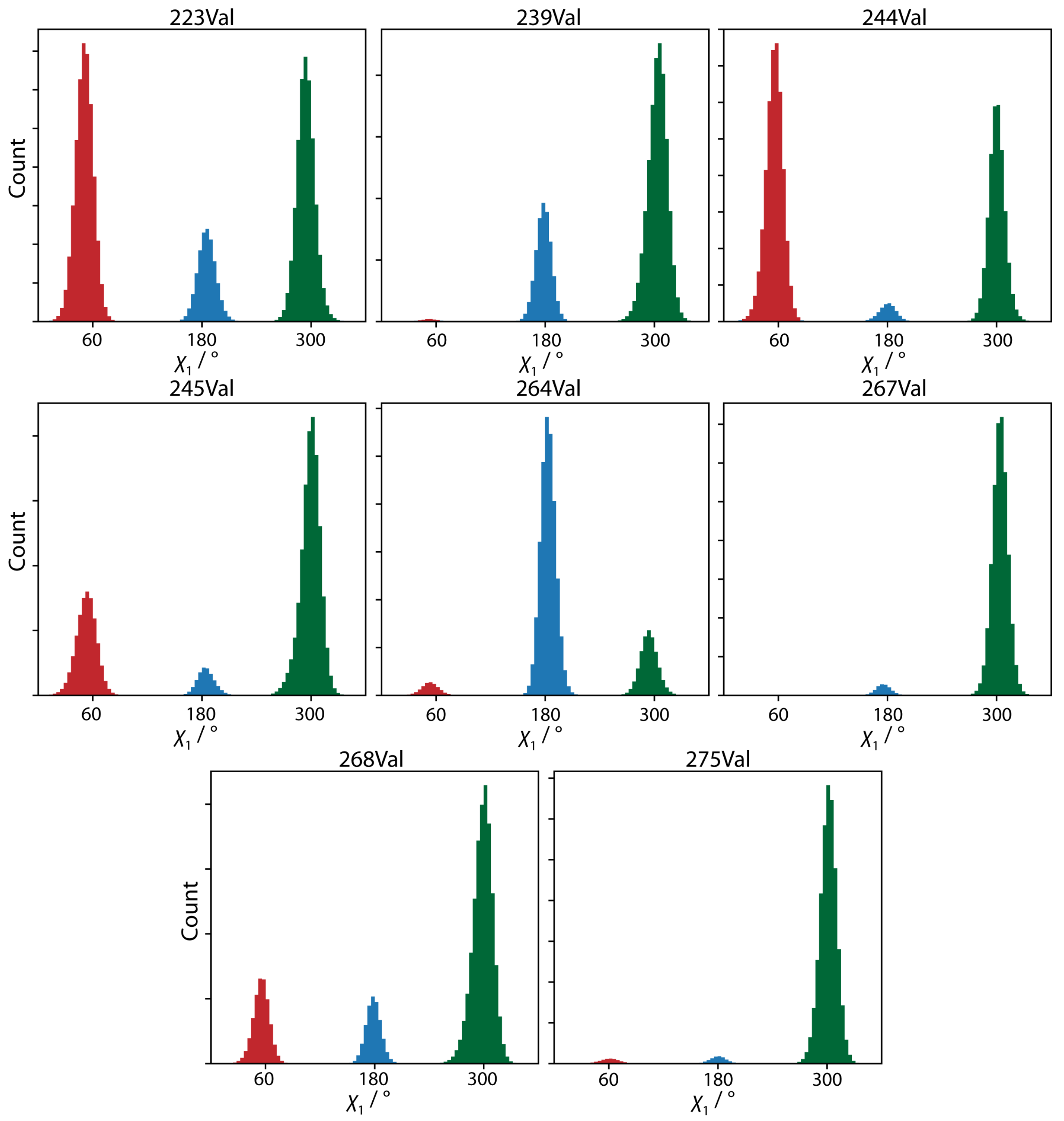

SI Figure 17. Histograms for the χ_1 angle of valine residues.

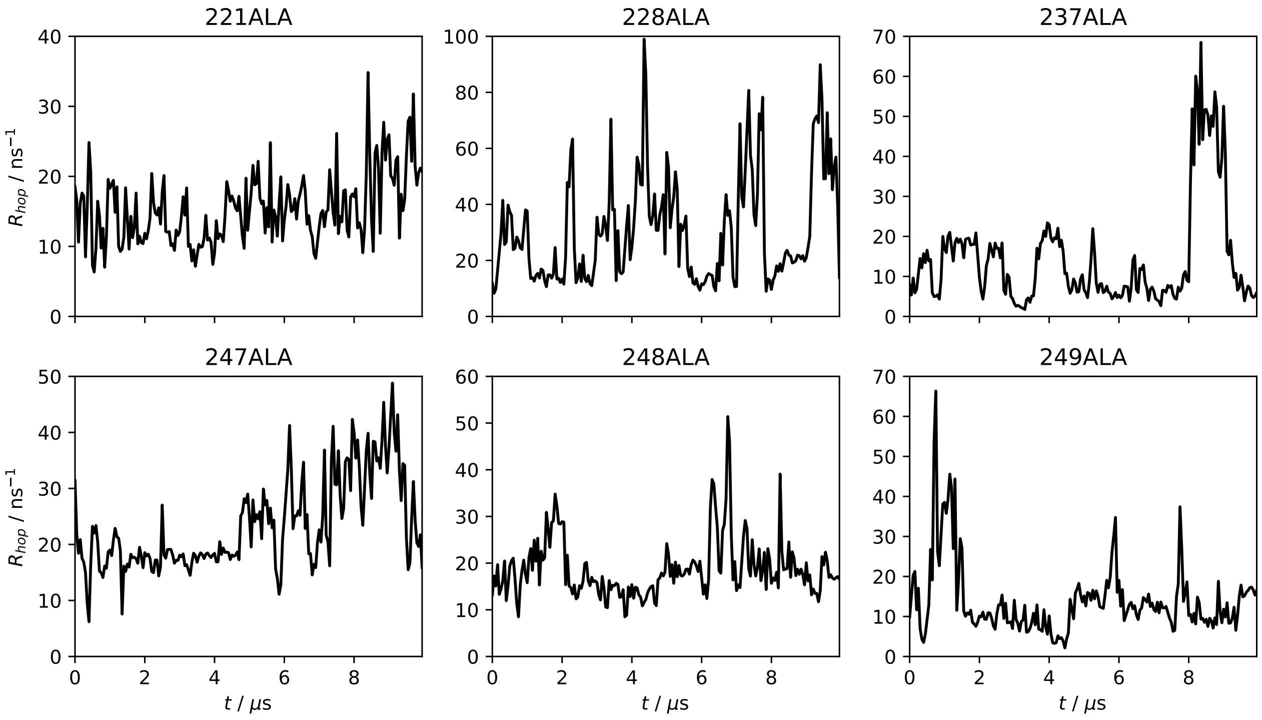

SI Figure 18. Binned methyl hopping rates for alanine.

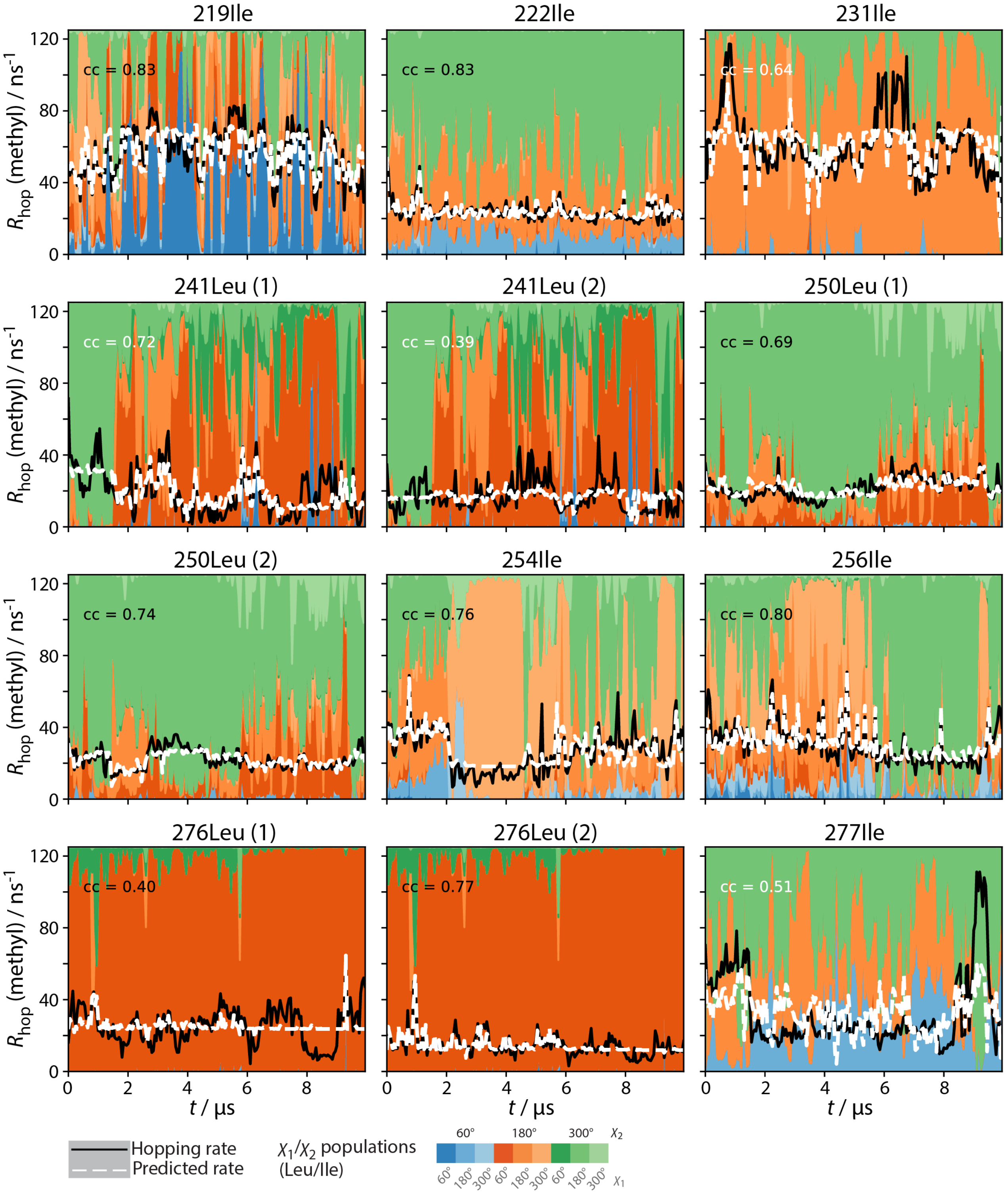

SI Figure 19. Binned methyl hopping rates for isoleucine and leucine.

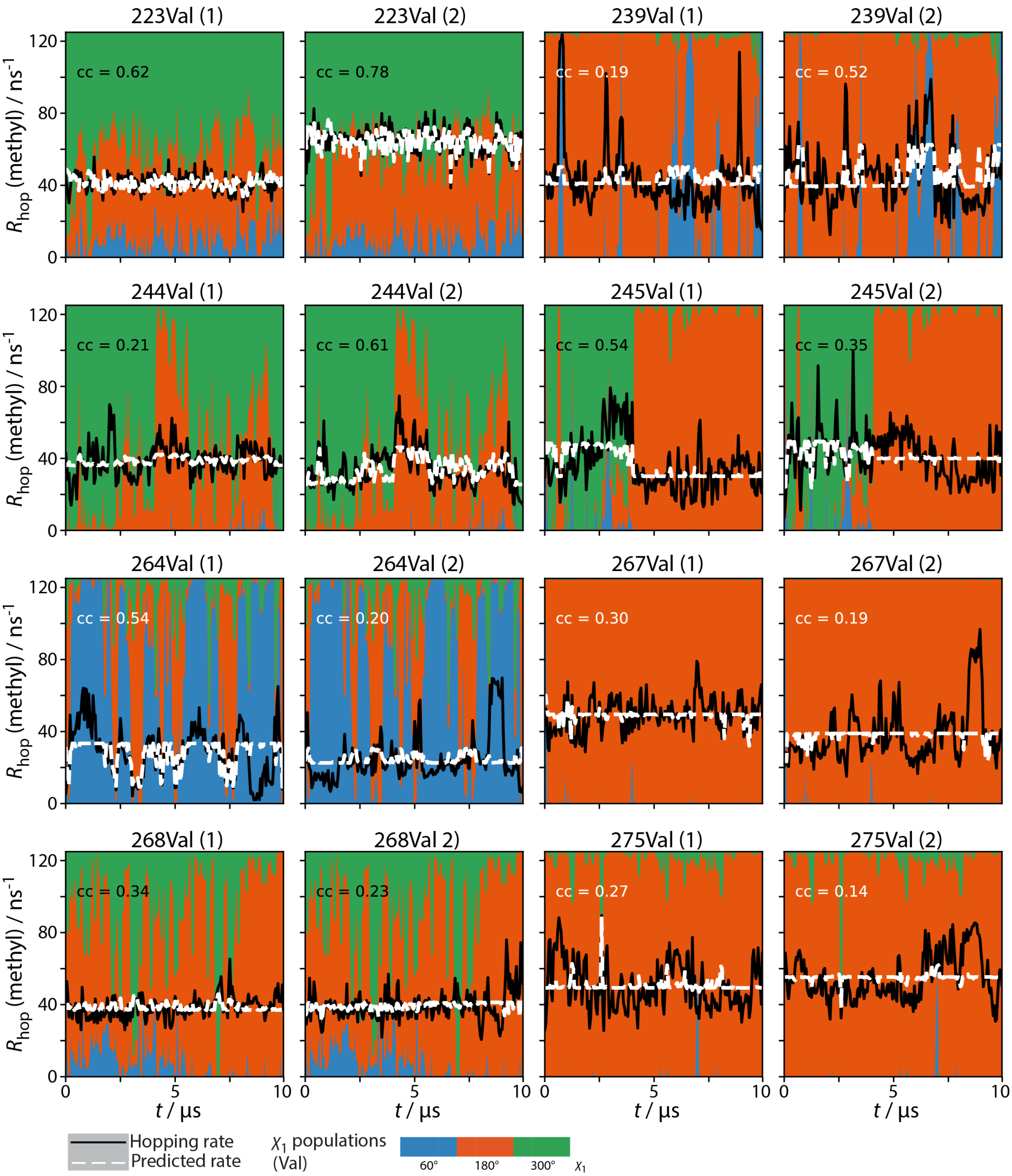

SI Figure 20. Binned methyl hopping rates for valine.

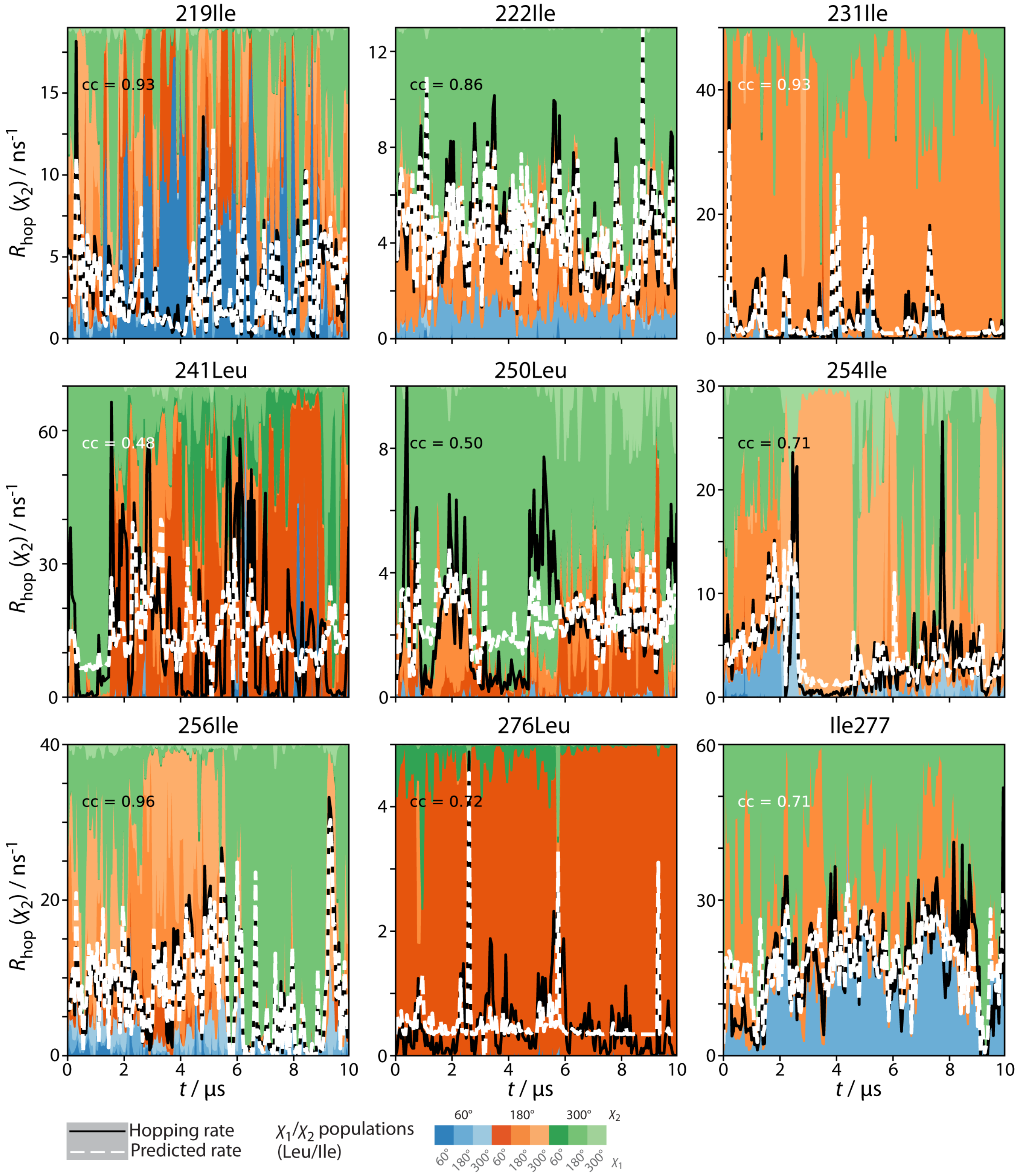

SI Figure 21. Binned χ_2 hopping rates.

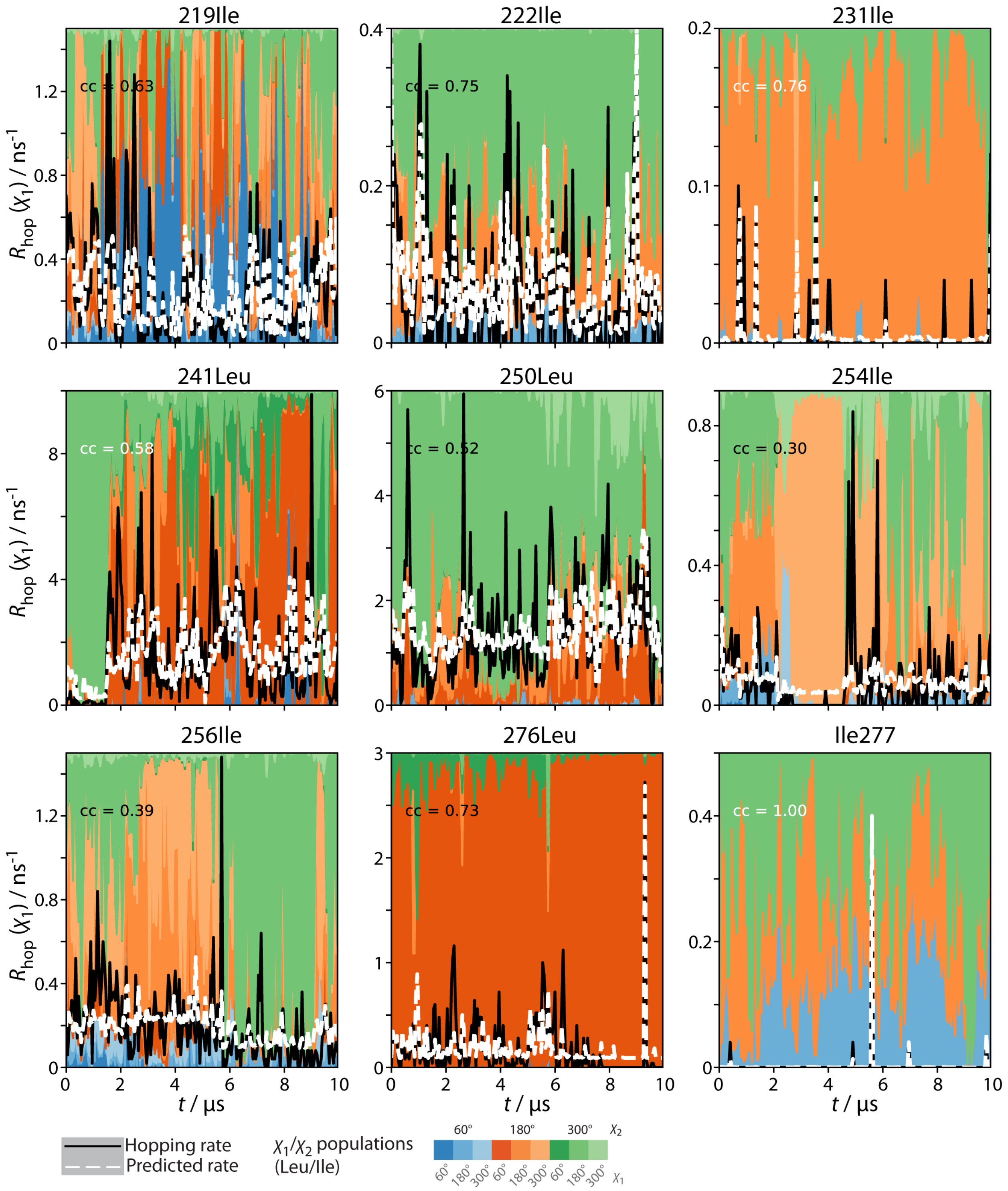

SI Figure 22. Binned χ_1 hopping rates.

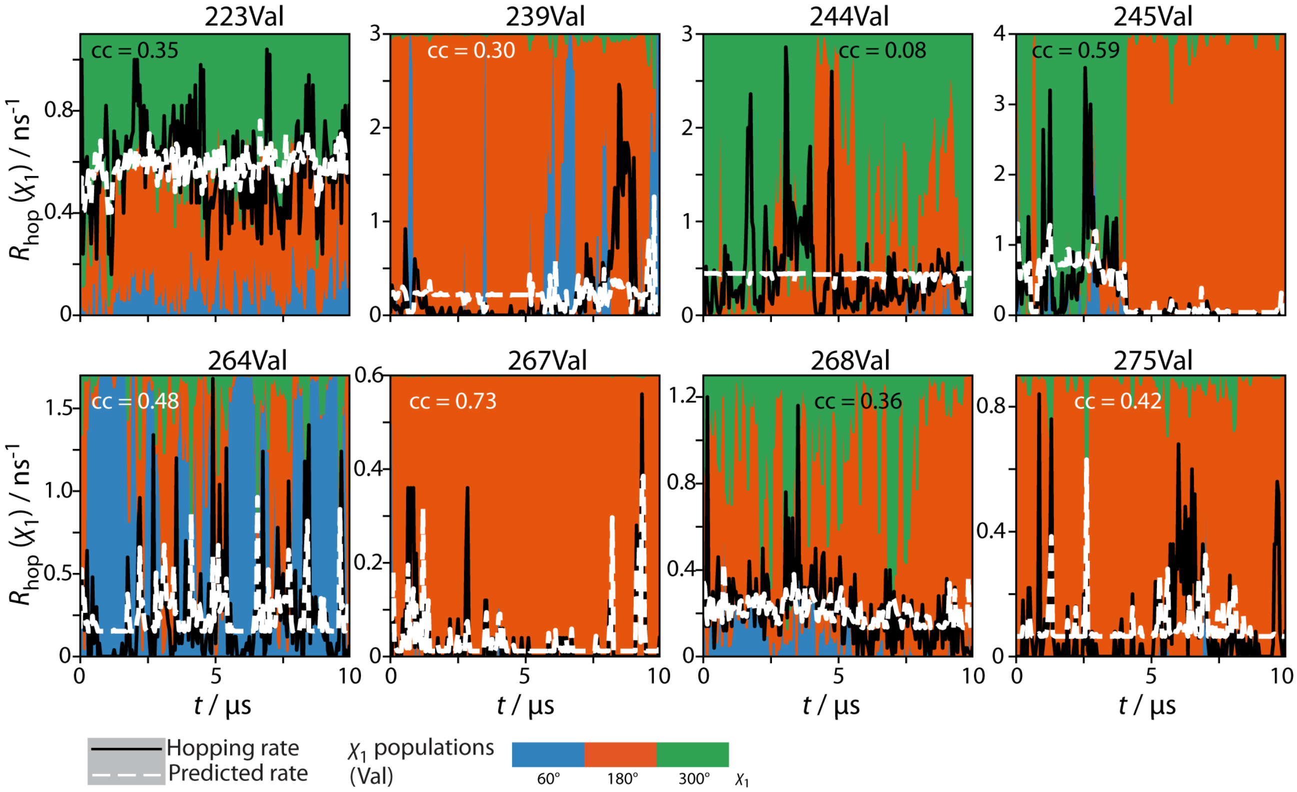

SI Figure 23. Binned χ_1 hopping rates.

12.Markov model construction and correlation function extraction

To produce SI Figure 18–SI Figure 23, it is necessary to assign the sidechains to a given rotameric state (here, we'll focus on the χ_1 and χ_2 states). Then, it is possible to construct a Markov model of the dynamics based on this assignment. For a given sidechain, we assign the state to be 0-2 or 0-8 (Val, Ile/Leu, respectively). Then, the Markov transition matrix is constructed such that if there are *N* frames in state *n*, and after one step, *K* of those frames transition to state *k*, then $T_{n\to k} = K/N$. Note that based on this definition, $\sum_k T_{n\to k} = 1$. Note that the choice of step size for the Markov model, τ, can be important in determining the Markov transition matrix, although in this case, we did not find significant changes in behavior when changing τ, and thus left it fixed to the minimum step size (5 ps). Then, if the system starts in a given ensemble of states, described by the vector, $\vec{\phi}$, then at a time, τ, later, the system should evolve to

$$\vec{\phi}_{t+\tau} = T \cdot \vec{\phi}_t \qquad \text{(S13)}$$

Then, if we have an exchange matrix, describing the transitions between states in the ensemble, we would similarly find that

$$\vec{\phi}_{t+\tau} = \exp K\tau \cdot \vec{\phi}_t \qquad \text{(S14)}$$

It follows that we may extract the exchange matrix from the transition matrix according to

$$\mathbf{K} = \log T / \tau \qquad \text{(S15)}$$

Note that this is the matrix logarithm, as opposed to the element-wise logarithm. It may be necessary to exclude some states from T, in case they are never populated, or if some transition brings the system into a given state, but never brings it out again.

From the exchange matrix, K, it is possible to extract the correlation function, as was previously detailed in ref. [18] (SI Section S5).

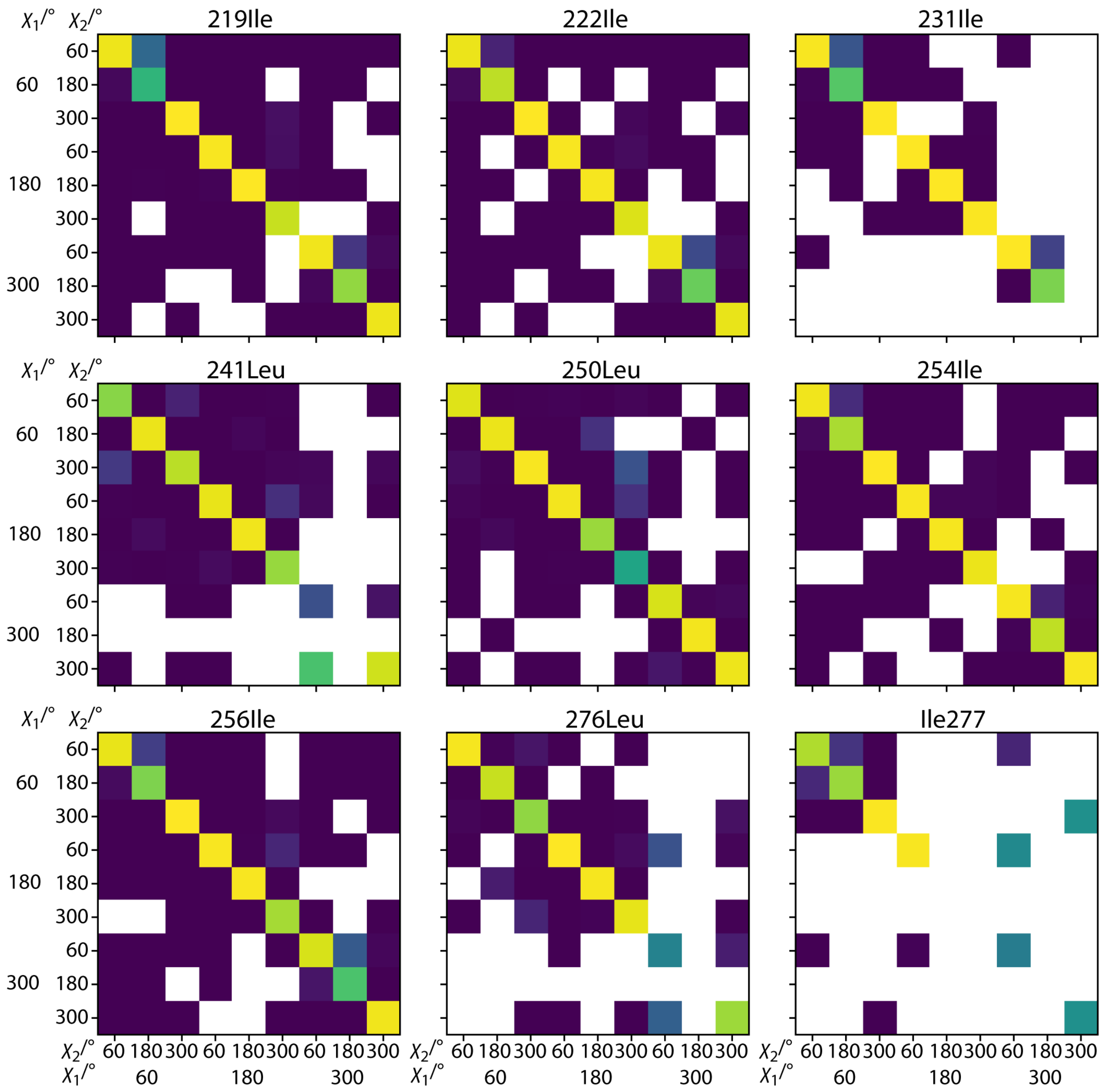

SI Figure 24. Markov matrices for Leu, Ile. The color indicates the probability of transitions, or the probability of staying in the same state (diagonal elements). White squares indicate that the given transition did not occur during the trajectory.

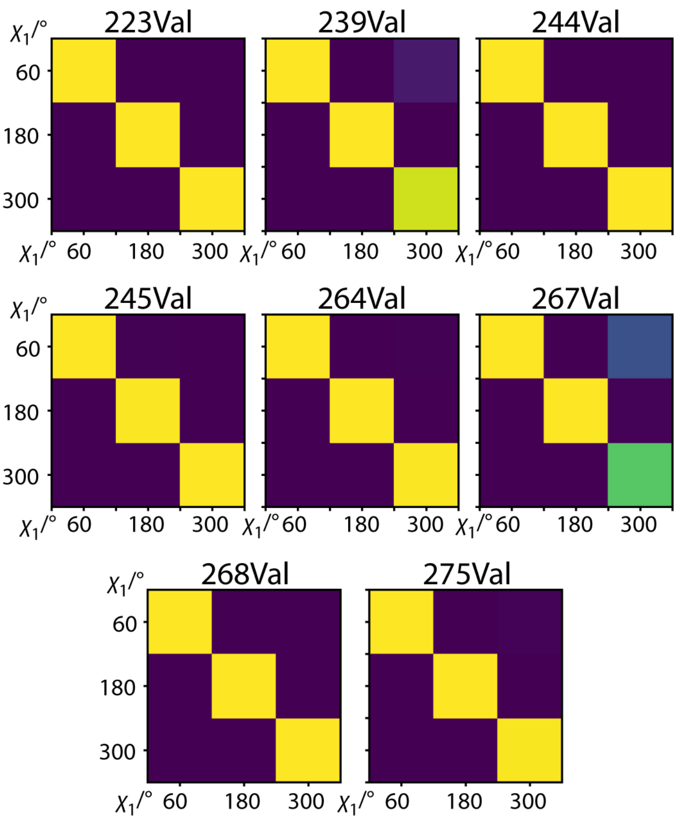

SI Figure 25. Markov matrices for Val. The color indicates the probability of transitions, or the probability of staying in the same state (diagonal elements). White squares indicate that the given transition did not occur during the trajectory.

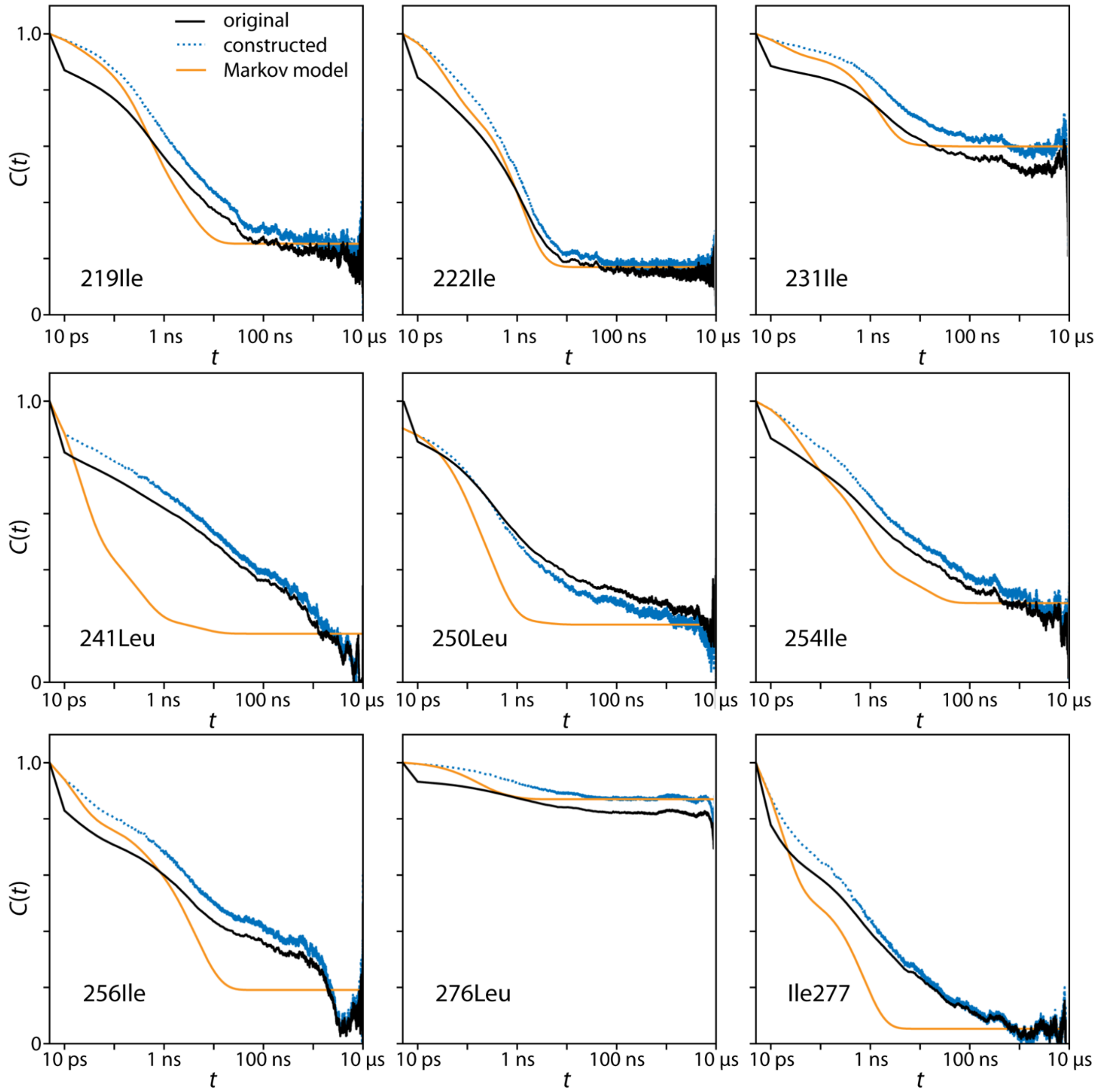

SI Figure 26. Markov-derived correlation functions for rotameric motion. Black, solid lines show the total reorientational correlation function for the Cγ–Cδ bond of Ile/Leu. Blue, dashed lines show the correlation function constructed from the χ_1/χ_2 states, effectively removing smaller amplitude librational motion. Orange solid lines show the correlation function reconstructed from a Markov model.

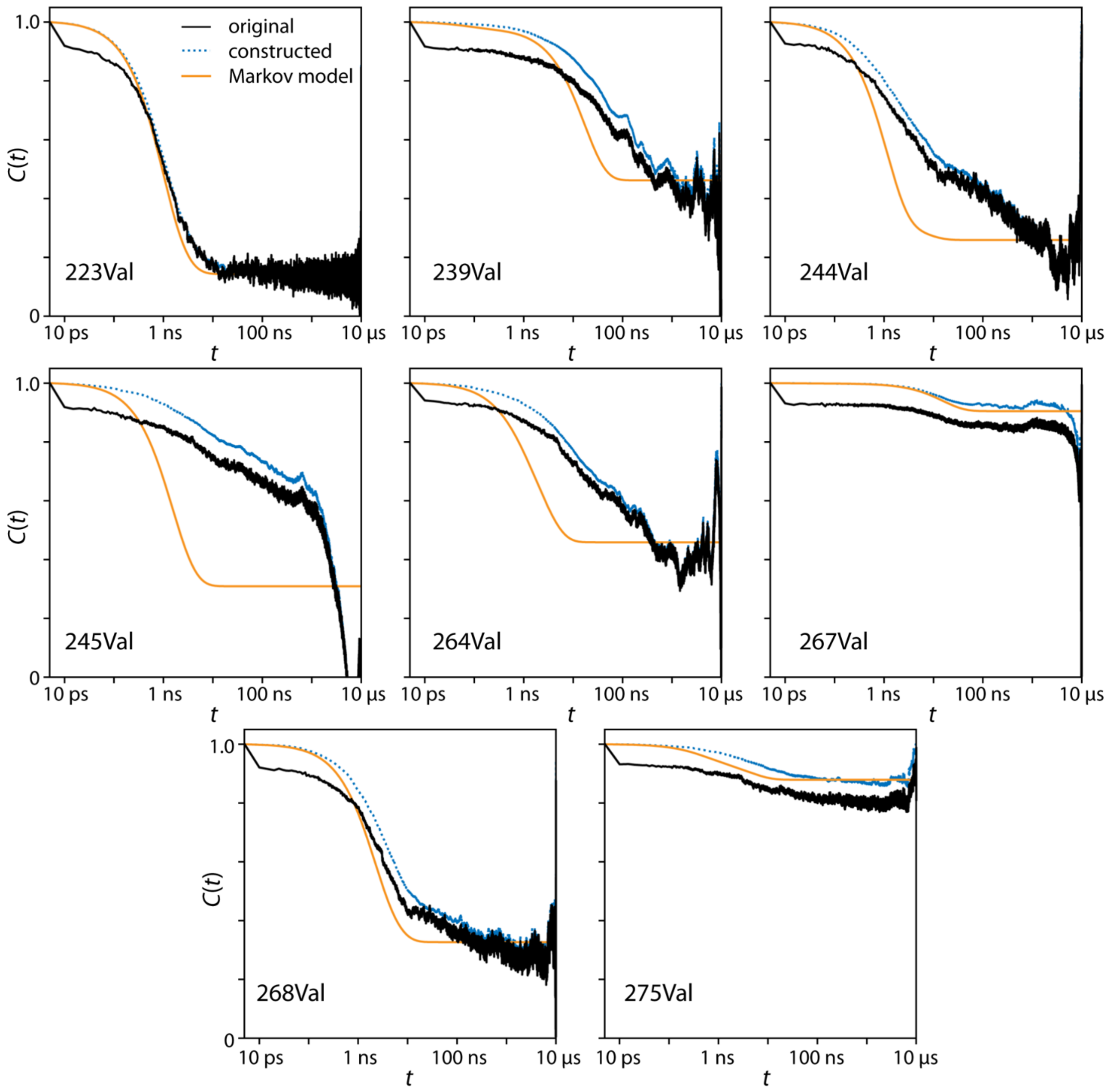

SI Figure 27. Markov-derived correlation functions for rotameric motion. Black, solid lines show the total reorientational correlation function for the Cβ–Cγ bond of Val. Blue, dashed lines show the correlation function constructed from the χ_1 states, effectively removing smaller amplitude librational motion. Orange solid lines show the correlation function reconstructed from a Markov model.

13. Entropy equilibration

In order to evaluate sidechain correlation based on entropy calculations, it is important that the side chain entropy is reasonably well-equilibrated. Here, we calculate the entropy as a function of trajectory length for the 10 µs trajectory (i.e., we calculate the entropy for only a part of the trajectory). While the entropy is not fully equilibrated, we see that the vast majority of the entropy of the simulation is indeed already obtained for the 10 µs trajectory.

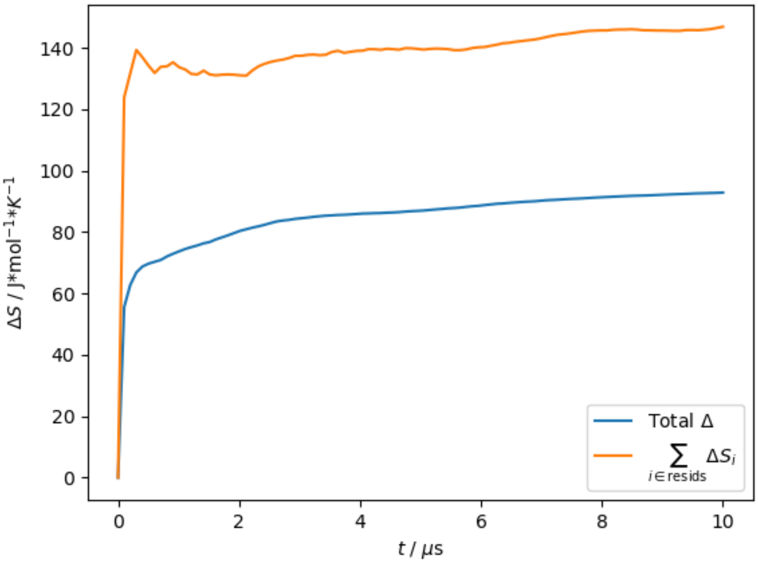

SI Figure 28. Total entropy and entropy summed over sidechains as a function of trajectory length.

14. Correlation of principle components with methyl dynamics

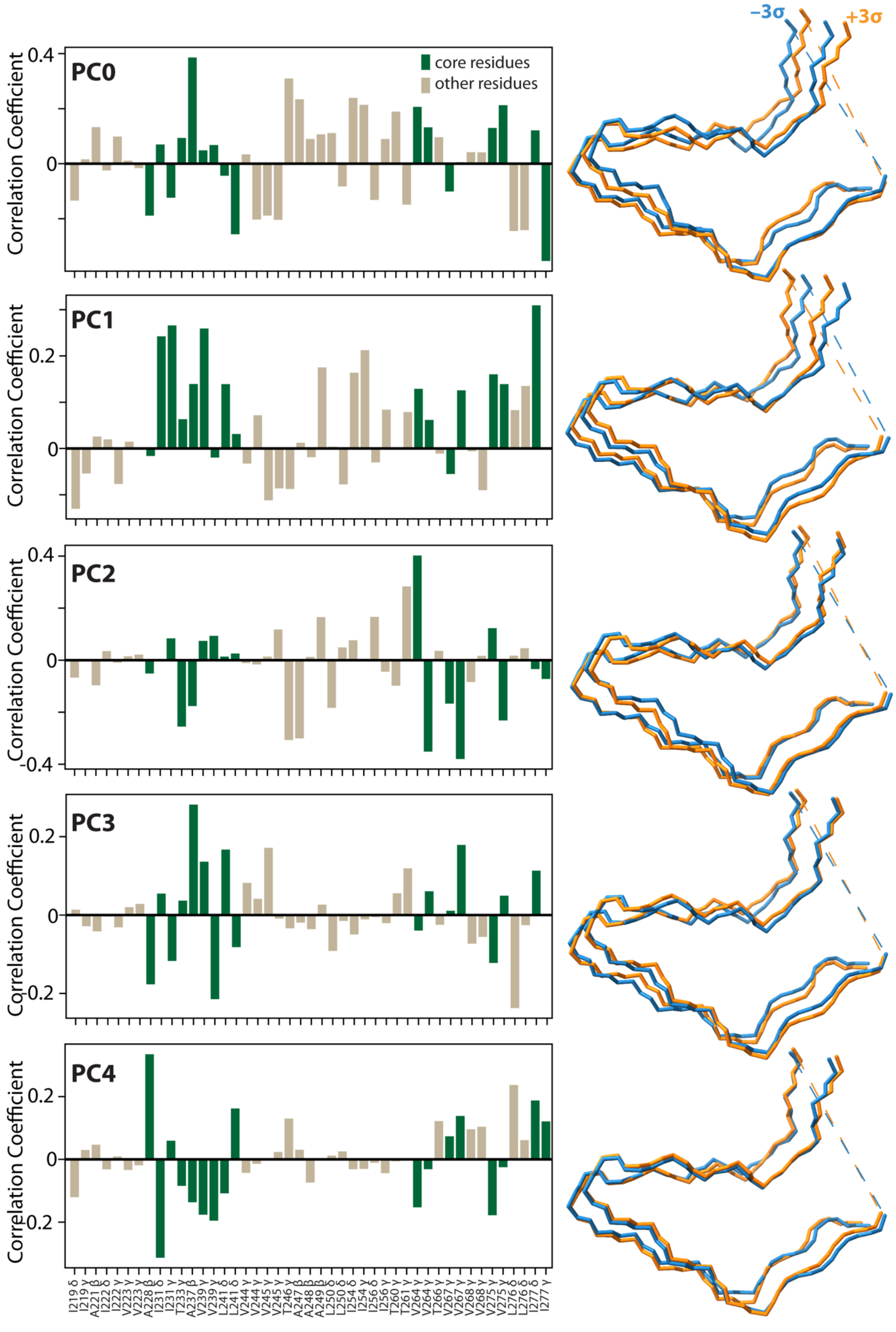

SI Figure 29. Methyl rotation vs. PCA modes 0-4.

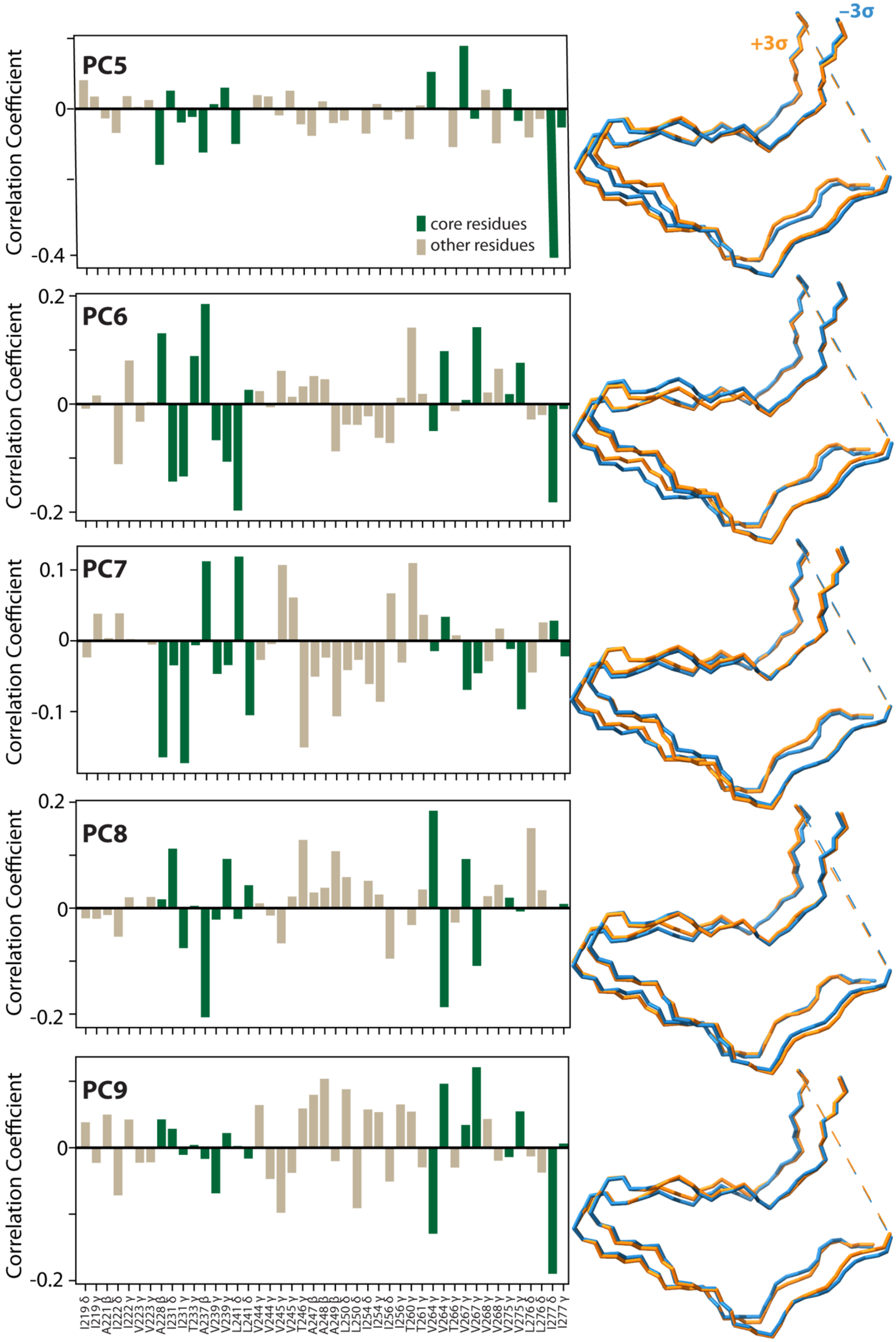

SI Figure 30. Methyl rotation vs. PCA modes 5-9.

15. References

(1) Munowitz, M. G.; Griffin, R. G.; Bodenhausen, G.; Huang, T. H. Two-Dimensional Rotational Spin-Echo Nuclear Magnetic Resonance in Solids: Correlation of Chemical Shift and Dipolar Interactions. *J. Am. Chem. Soc.* **1981**, *103* (10), 2529–2533. https://doi.org/10.1021/ja00400a007.

(2) Smith, A. A.; Testori, E.; Cadalbert, R.; Meier, B. H.; Ernst, M. Characterization of Fibril Dynamics on Three Timescales by Solid-State NMR. *J. Biomol. NMR* **2016**, *65* (3), 171–191. https://doi.org/10.1007/s10858-016-0047-8.

(3) Smith, A. A. INFOS: Spectrum Fitting Software for NMR Analysis. *J. Biomol. NMR* **2017**, *67* (2), 77–94. https://doi.org/10.1007/s10858-016-0085-2.

(4) Smith, A. A.; Zumpfe, K. pyDIFRATE, 2023. https://alsinmr.github.io/pyDR.

(5) Efron, B. *The Jackknife, the Bootstrap and Other Resampling Plans*; Society for Industrial and Applied Mathematics, 1982. https://doi.org/10.1137/1.9781611970319.

(6) Berendsen, H. J. C.; van der Spoel, D.; van Drunen, R. GROMACS: A Message-Passing Parallel Molecular Dynamics Implementation. *Comput. Phys. Commun.*, 1995, *91*, 43–56. https://doi.org/10.1016/0010-4655(95)00042-E.

(7) Abraham, M. J.; Murtola, T.; Schulz, R.; Páll, S.; Smith, J. C.; Hess, B.; Lindahl, E. GROMACS: High Performance Molecular Simulations through Multi-Level Parallelism from Laptops to Supercomputers. *SoftwareX*, 2015, *1–2*, 19–25. https://doi.org/10.1016/j.softx.2015.06.001.

(8) Wasmer, C.; Lange, A.; Van Melckebeke, H.; Siemer, A. B.; Riek, R.; Meier, B. H. Amyloid Fibrils of the HET-s(218-289) Prion Form a β Solenoid with a Triangular Hydrophobic Core. *Science*, 2008, *319*, 1523–1526. https://doi.org/10.1126/science.1151839.

(9) Van Melckebeke, H.; Wasmer, C.; Lange, A.; AB, E.; Loquet, A.; Böckmann, A.; Meier, B. H. Atomic-Resolution Three-Dimensional Structure of HET-s(218-289) Amyloid Fibrils by Solid-State NMR Spectroscopy. *J. Am. Chem. Soc.*, 2010, *132*, 13765–13775. https://doi.org/10.1021/ja104213j.

(10) Lange, A.; Gattin, Z.; Van Melckebeke, H.; Wasmer, C.; Soragni, A.; Gunsteren, W. F.; Meier, B. H. A Combined Solid-State NMR and MD Characterization of the Stability and Dynamics of the HET-s(218-289) Prion in Its Amyloid Conformation. *ChemBioChem*, 2009, *10*, 1657–1665. https://doi.org/10.1002/cbic.200900019.

(11) Jorgensen, W. L.; Chandrasekhar, J.; Madura, J. D.; Impey, R. W.; Klein, M. L. Comparison of Simple Potential Functions for Simulating Liquid Water. *J. Chem. Phys.*, 1983, *79*, 926–935. https://doi.org/10.1063/1.445869.

(12) Berendsen, H. J. C.; Grigera, J. R.; Straatsma, T. P. The Missing Term in Effective Pair Potentials. *J. Phys. Chem.* **1987**, *91* (24), 6269–6271. https://doi.org/10.1021/j100308a038.

(13) Lindorff-Larsen, K.; Piana, S.; Palmo, K.; Maragakis, P.; Klepeis, J. L.; Dror, R. O.; Shaw, D. E. Improved Side-Chain Torsion Potentials for the Amber ff99SB Protein Force Field. *Proteins*, 2010, *78*, 1950–1958. https://doi.org/10.1002/prot.22711.

(14) Hoffmann, F.; Xue, M.; Schafer, L. V.; Mulder, F. A. A. Narrowing the Gap between Experimental and Computational Determination of Methyl Group Dynamics in Proteins. *Phys Chem Chem Phys*, 2018, *20*, 24577–24590. https://doi.org/10.1039/c8cp03915a.

(15) Hoffmann, F.; Mulder, F. A. A.; Schäfer, L. V. Accurate Methyl Group Dynamics in Protein Simulations with AMBER Force Fields. *J. Phys. Chem. B* **2018**, *122* (19), 5038–5048. https://doi.org/10.1021/acs.jpcb.8b02769.

(16) Smith, A. A.; Ernst, M.; Riniker, S.; Meier, B. H. Localized and Collective Motions in HET-s(218-289) Fibrils from Combined NMR Relaxation and MD Simulation. *Angew. Chem. Int. Ed.*, 2019, *58*, 9483–9488. https://doi.org/10.1002/ange.201901929.

(17) Smith, A. A. Interpreting NMR Dynamic Parameters via the Separation of Reorientational Motion in MD Simulation. *J. Magn. Reson. Open* **2022**, *10–11*, 100045. https://doi.org/10.1016/j.jmro.2022.100045.

(18) Zumpfe, K.; Smith, A. A. Model-Free or Not? *Front. Mol. Biosci.* **2021**, *8*, 727553. https://doi.org/10.3389/fmolb.2021.727553.
